# MCL-1 inhibition provides a new way to suppress breast cancer metastasis and increase sensitivity to dasatinib

**DOI:** 10.1186/s13058-016-0781-6

**Published:** 2016-12-08

**Authors:** Adelaide I. J. Young, Andrew M. K. Law, Lesley Castillo, Sabrina Chong, Hayley D. Cullen, Martin Koehler, Sebastian Herzog, Tilman Brummer, Erinna F. Lee, Walter D. Fairlie, Morghan C. Lucas, David Herrmann, Amr Allam, Paul Timpson, D. Neil Watkins, Ewan K. A. Millar, Sandra A. O’Toole, David Gallego-Ortega, Christopher J. Ormandy, Samantha R. Oakes

**Affiliations:** 1 Cancer Research Division, Garvan Institute of Medical Research and the Kinghorn Cancer Centre, 384 Victoria Street, Darlinghurst, NSW 2010 Australia; 2 Centre for Biological Systems Analysis (ZBSA) and Centre for Biological Signallling Studies, Albert-Ludwigs-University, Stefan-Meier-Strasse 17, 79104 Freiburg, Germany; 3 Spemann Graduate School for Biology and Medicine and Faculty of Biology, Albert-Ludwigs-University, Stefan-Meier-Strasse 17, 79104 Freiburg, Germany; 4 BIOSS Centre for Biological Signalling Studies, Albert-Ludwigs-University Freiburg, Schänzlestrasse 18, 79104 Freiburg, Germany; 5 Olivia Newton-John Cancer Research Institute, 145 Studley Rd, Heidelberg, Victoria 3084 Australia; 6 School of Cancer Medicine and Department of Chemistry and Physics, La Trobe University, Melbourne, Victoria 3086 Australia; 7 The Walter and Eliza Hall Institute of Medical Research, 1G Royal Parade, Parkville, Victoria 3052 Australia; 8 Department of Medical Biology, The University of Melbourne, Parkville, Victoria 3010 Australia; 9 St. Vincent’s Clinical School, UNSW Medicine, Victoria Street, Darlinghurst, NSW 2052 Australia; 10 Department of Anatomical Pathology, South Eastern Area Laboratory Service, St George Hospital, Grey St, Kogarah, NSW 2217 Australia; 11 Sydney Medical School, Sydney University, Fisher Rd, Camperdown, NSW 2006 Australia; 12 Department of Tissue, Pathology and Diagnostic Oncology, Royal Prince Alfred Hospital, Missenden Rd, Camperdown, 2050 NSW Australia

**Keywords:** Myeloid cell leukemia-1, BIMs2A, Metastasis, Invasion, Breast cancer, BH3 mimetics, Cofilin, SRC family kinase

## Abstract

**Background:**

Metastatic disease is largely resistant to therapy and accounts for almost all cancer deaths. Myeloid cell leukemia-1 (MCL-1) is an important regulator of cell survival and chemo-resistance in a wide range of malignancies, and thus its inhibition may prove to be therapeutically useful.

**Methods:**

To examine whether targeting MCL-1 may provide an effective treatment for breast cancer, we constructed inducible models of BIMs2A expression (a specific MCL-1 inhibitor) in MDA-MB-468 (MDA-MB-468-2A) and MDA-MB-231 (MDA-MB-231-2A) cells.

**Results:**

MCL-1 inhibition caused apoptosis of basal-like MDA-MB-468-2A cells grown as monolayers, and sensitized them to the BCL-2/BCL-XL inhibitor ABT-263, demonstrating that MCL-1 regulated cell survival. In MDA-MB-231-2A cells, grown in an organotypic model, induction of BIMs2A produced an almost complete suppression of invasion. Apoptosis was induced in such a small proportion of these cells that it could not account for the large decrease in invasion, suggesting that MCL-1 was operating via a previously undetected mechanism. MCL-1 antagonism also suppressed local invasion and distant metastasis to the lung in mouse mammary intraductal xenografts. Kinomic profiling revealed that MCL-1 antagonism modulated Src family kinases and their targets, which suggested that MCL-1 might act as an upstream modulator of invasion via this pathway. Inhibition of MCL-1 in combination with dasatinib suppressed invasion in 3D models of invasion and inhibited the establishment of tumors in vivo.

**Conclusion:**

These data provide the first evidence that MCL-1 drives breast cancer cell invasion and suggests that MCL-1 antagonists could be used alone or in combination with drugs targeting Src kinases such as dasatinib to suppress metastasis.

**Electronic supplementary material:**

The online version of this article (doi:10.1186/s13058-016-0781-6) contains supplementary material, which is available to authorized users.

## Background

Metastatic disease remains largely incurable and accounts for almost all cancer deaths. Metastasis is a multistep process where primary tumor cells escape and colonize distant organs and tissues. There are several steps that lead to the acquisition of this lethal phenotype, which include epithelial to mesenchymal transition, cytoskeletal remodeling and cellular movement, intravasation, survival in the vasculature, extravasation and the acquisition of cellular properties of the distant niche. Importantly, cell survival underlies every stage of this process [1]. Myeloid cell leukemia-1 (MCL-1) is a potent survival factor for normal and malignant tissues and is associated with chemo-resistance in a wide range of tumor types [2]. For this reason, there is great interest in the development of small molecule pharmaceuticals that inhibit MCL-1, with some compounds showing high specificity and potent anti-tumor effects in vitro [3, 4], but the poor pharmacokinetics of these compounds limits their use in vivo.

MCL-1 is a member of the BCL-2 family of proteins that regulate the intrinsic (mitochondrial) apoptotic cascade [5]. MCL-1 interacts with the pro-apoptotic BCL-2 family members and protects normal and malignant cells from undergoing programmed cell death. The pro-apoptotic family members include two subgroups: the BH3-only sensor proteins (e.g., BIM, NOXA and PUMA), which trigger the intrinsic apoptotic cascade in response to cytotoxic insults or cellular stresses; and BAX and BAK, the apoptotic effectors [5]. Following a death stimulus, BAX and BAK change conformation and oligomerize, leading to outer mitochondrial membrane permeabilization and the release of apoptogenic factors, leading eventually to cell death. MCL-1 is more than just a BCL-2-like survival factor [2]. MCL-1 is anti-apoptotic at the outer mitochondrial membrane, but can localize to the inner mitochondrial membrane, where it regulates oxidative phosphorylation and the maintenance of mitochondrial structure [6]. MCL-1 can also interact with CDK1, PCNA and CHK1 in the nucleus, where it regulates cell cycle progression and DNA damage [7]. Hence MCL-1 is a protein with multiple functions in mitochondrial-dependent cell death machinery, metabolic oxidative phosphorylation and DNA damage responses.

The *MCL-1* gene is one of the most frequent focal amplifications in breast cancer, occurring in approximately 30% of cases [8]. High *MCL-1* expression has been found to correlate with poor prognosis in mixed breast cancers [9] and de-novo copy number amplification correlates with therapeutic resistance [8–12]. MCL-1 is a key player in resistance to a wide range of therapies [9, 11, 13]. MCL-1 protein is observed in most breast cancer subtypes [14]. MCL-1 also has been shown to confer the survival of breast cancer cells in vitro [4]. These data suggest that MCL-1 could provide a therapeutic target for a wide range of breast cancer patients.

Here, we have modeled MCL-1 antagonism in breast cancer cell lines by inducible expression of a modified form (L62A/F69A double mutant) of the short isoform of BIM (BIMs2A/2A), which mimics the actions of a highly specific small molecule antagonist [15]. This genetic approach was chosen because it was effective in models of acute myeloid leukemia and can be precisely controlled using inducible vector systems [16, 17]. BIMs2A acts similarly to NOXA because it binds preferentially and with high affinity to the hydrophobic pocket of MCL-1, thereby releasing bound BH3-only proteins and blocking engagement with activated BAX/BAK. Unlike NOXA and knockdown strategies, BIMs2A binds and disrupts the interactions of MCL-1, while maintaining its stability. The effects of this antagonist on cell death, invasion and metastasis were examined in vitro, using traditional culture techniques and a novel model of cell invasion, and in vivo using cell lines grown as intraductal xenografts, a technique that recapitulates the requirement for cancer cells to breach the basement membrane of the mammary duct to metastasize.

## Methods

Additional materials and methods are provided in Additional file 1.

### Mice

Immune-compromised NODScidIL2gamma^–/–^ mice were housed in SPF conditions in a 12-hour:12-hour light:dark cycle and given food and water ad libitum. Doxycycline (DOX)-containing food (700 mg/kg) was purchased from Gordon’s Specialty Stock Feeds and replaced weekly. Intraductal injections were modified from a previously described protocol without a Y incision in the abdomen [18]. For longitudinal studies, mice were randomized into DOX-treated or control-treated groups and monitored twice weekly for tumor growth, and measurements were taken until an ethical end point of 10% tumor burden or prior if the animal succumbed to tumor/metastasis-induced morbidity. For cross-sectional studies, mice were again randomized into DOX-treated or control-treated groups and sacrificed at 9 weeks (MDA-MB-231-2A xenografts) or 12 weeks (MDA-MB-468-2A xenografts) post tumor cell inoculation. For tail vein injections, mice were injected (with 1,500,000 MDA-MB-231-2A cells or with 2,000,000 MDA-MB-468-2A and MDA-MB-157 cells) using a 100 μl injection into the dorsal tail vein before harvest at 9 weeks post injection. At the end of the experiment, as indicated in the figures, mice were euthanized with CO_2_ asphyxiation and the mammary glands, tumor and lungs were harvested and fixed for 4 hours in 10% buffered formalin at room temperature. Where possible, mammary glands were whole mounted and tissues were processed for histology as described previously [19]. After fixation, the mammary glands, tumors or lungs were sectioned and either stained with hematoxylin and eosin for routine histochemistry or stained with BIM (CST 2933), high molecular weight cytokeratin (Leica 34BE12), Vimentin (Leica NCL-L-VIM-V9), MCL-1 (ThermoScience MA5-13932), Cleaved Caspase-3 (CST ASP175 9664), multi-cytokeratin (Leica C-11) and Ki67 (ThermoScientific SP6) using DAKO immunohistochemistry as per the manufacturer’s instructions. All sections from tumors and lungs in each model were cut, sectioned, retrieved and stained at the same time permitted with each antigen.

### 2D and 3D in-vitro experiments

Pools of BIMs2A or empty vector (EV) cells were made by routine cloning into an all-in-one tetracycline inducible vector (SH570MK as detailed in Additional file 1﻿) and selected using Puromycin. BIMs2A expression was induced with 2 μg/ml DOX or vehicle control daily in the media and cells were harvested at the time points indicated in the figures. Annexin V PI staining was performed using the Annexin V-FITC Apoptosis Kit (Biovision, CA, USA) as per the manufacturer’s instructions. ABT-263 (5 μg/ml) was added to the media at the indicated times. For siRNA experiments, 5 nM siRNAs targeting *MCL-1* (DHA-L-004384-00-0005) or non-targeting control siRNA (D-001206-14-05) was premixed with RNAiMAX (ThermoFisher) and cells were transfected the day after plating at a density of 1 × 10^5^ cells per well with ON-TARGETplus SmartPools of MCL-1 or nontargeting controls siRN﻿A﻿s as per the manufacturer’s instructions. A1210477 and UMI-77 (Selleckchem, MA, USA) was added to the media at 5 or 10 μM respectively as indicated in the figures. Dasatinib (Bristol-Myers Squibb, Princeton, NJ, USA) was added to the media in 2D and 3D at a concentration of 1 μM and 200 nM respectively. 3D collagen I/fibroblast models were performed as described previously [20].

### Human invasive carcinoma breast tissue microarrays

Immunohistochemistry was used to assay MCL-1 protein expression using a mouse monoclonal antibody to MCL-1 (ThermoFischer (Pierce) MA5-13932) on TMAs constructed from tumors from a cohort of 292 patients diagnosed with invasive ductal breast carcinoma described in [21]. MCL-1 protein could only be detected in a subset of 246 of these cases due to missing or folded cores. The cohort consists of cases of invasive ductal carcinoma of no special type, median age 54 (range 24–87), with a median follow-up of 64 months (range 0–152.1). Of these, 68.6% were ER+, 57.1% were PR+, 18.7% were HER-2 amplified (by FISH) and 43.3% were lymph node-positive. Endocrine therapy (TAM) was given to 49.3% of patients and chemotherapy (AC or CMF) to 38%.

### Polyacrylamide gel electrophoresis, immunoprecipitation and western blotting

Immunoprecipitates were made using antibodies to MCL-1 (ThermoScience MA5-13932), BCL-2 (Millipore 05-729) and BCL-XL (CST 2764) using the TrueBlot kit according to the manufacturer’s instructions (Rockland Immunochemicals). Then 20 μg reduced protein was loaded in each well of 12% NuPAGE SDS polyacrylamide gels (Life Technologies) and separated using electrophoresis. Proteins were transferred to Immun-Blot PVDF (Biorad) and analyzed by western blot for mouse MCL-1 (CST 5453), BCL-2 (Millipore 05-729), BIM (CST 2933), PUMA (CST 4976), NOXA (ENZO ALX-804-C10), p53 (Epitomics 1026-1), Cofilin (total) (CST 5175), S3-Cofilin (CST 3313) and beta-ACTIN (Santa Cruz AC-74, A5316).

### 3D organotypic collagen I/fibroblast invasion assay

The production of contracted matrices is described elsewhere [20] and detailed methods are provided in Additional file 1. Contracted matrices were seeded with 1 × 10^5^ MDA-MB-231-2A cells and allowed to grow for 4 days, mounted on a metal grid and raised to an air–liquid interface to initiate invasion, which resulted in the matrix being fed from below with the media supplemented with either vehicle, DOX, dasatinib or a combination of DOX and dasatinib commencing at seeding (day 1) or 5 days after seeding (day 5). All treatments were performed on three independent matrices. Cells were allowed to invade for a total of 10 days towards the chemo-attractive gradient created by the air–liquid interface and then harvested for immunohistochemistry for multi-cytokeratin (Leica-Novocastra C-11) (invasion), Ki67 (ThermoScientific SP6) (proliferation) or Cleaved Caspase 3 (Cell Signaling Asp175 5A) (apoptosis) and scored as detailed in Additional file 1.

### Image analysis and statistical analysis

Quantification of the number and size of metastases (using sections stained with an antibody against human-Vimentin) and BIM intensity (using an antibody raised against human BIM) was performed using macros designed using FIJI image analysis software (http://fiji.sc/Fiji) that are available from the corresponding author and are described in Additional file 1. Chi-squared analysis, Kaplan–Meier survival analysis and univariate analysis (UVA) using the Cox proportional hazards model were performed to determine correlations between MCL-1, clinicopathological features and outcome performed in Statview SE. All other data and statistics were analyzed in Prism6 for MacOSX. Data were graphed and parametric or nonparametric tests, as indicated in the figure legends, were used for normally distributed and skewed data respectively, and statistically significant groups were determined using bars as shown in the figures.

## Results

### MCL-1 is widely expressed in breast cancer cell lines

**Fig. 1:**
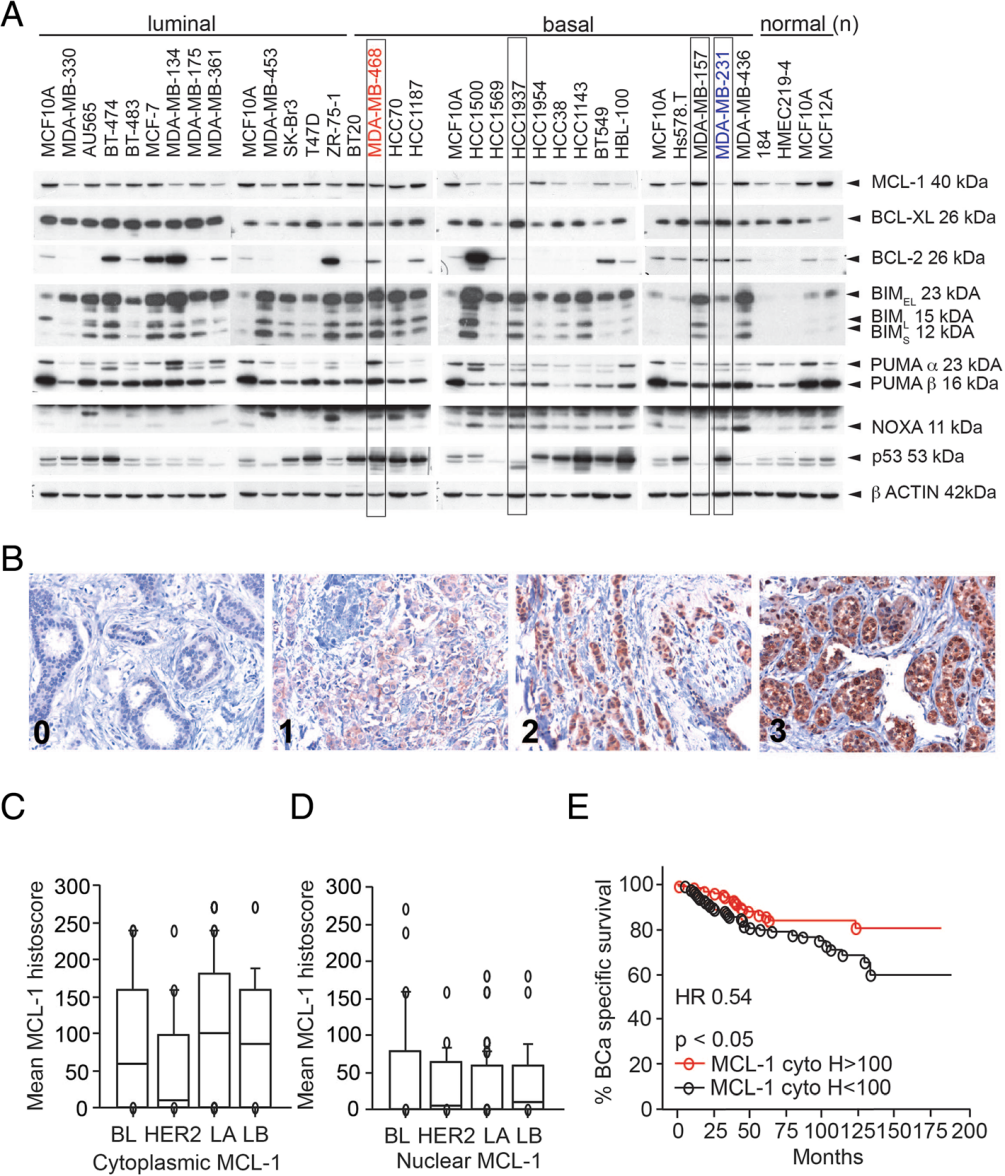
Levels of MCL-1, BCL-XL, BCL-2, BIM, PUMA, NOXA and p53 in breast cancer cell lines, immortalized breast epithelial cell lines and breast carcinoma. (**a**) Western blot using breast cell lines. Lysates from MCF10A cell lines were loaded in the first lane of each gel as a control for exposure and β-actin was used as a loading control. Data are representative of three independent western blots. (**b**) Immunohistochemistry using an antibody to MCL-1 in breast carcinoma tissue microarrays showing absent to strong (*0–3*) cytoplasmic levels. Nuclear staining was also detected in some cases (*3*). Box and whisker plots depicting the mean Histoscore of (**c**) cytoplasmic and (**d**) nuclear MCL-1 staining in basal-like (*BL*), HER2-positive (*HER2*), luminal A (*LA*) and luminal B (*LB*) breast carcinoma tissue microarrays. (**e**) Kaplan–Meier survival curve for breast cancer specific survival of luminal A patients with higher cytoplasmic MCL-1 (Histoscore > 100) or lower cytoplasmic MCL-1 (Histoscore > 100). Log-rank *p* value. *HR* hazard ratio, *MCL-1* myeloid cell leukemia 1 (Color figure online)

We investigated the levels of MCL-1 and the other BCL-2 family members BCL-2, MCL-1, BIM, PUMA and NOXA in 32 human breast cancer and immortalized breast epithelial cell lines (Fig. 1a). Variable levels of MCL-1 and BCL-XL were detected in all cell lines. BCL-2 levels were more variable, with only a small proportion (5/32, 15%) displaying high expression. MCL-1 was present in 17 cell lines with low or zero levels of BCL-2 (Fig. 1a). The MCL-1-interacting BH3-only proteins BIM, PUMA and NOXA were expressed at varying levels in all cell lines tested (Fig. 1a).

### MCL-1 protein was widely expressed by invasive breast carcinomas independently of subtype and was predictive of overall survival

We investigated the importance of *MCL-1 *copy number variations and expression in breast tumors using publically available datasets. Gains and amplifications in *MCL-1 *copy number were observed in 14% of all cases in the TCGA dataset (CBioPortal [22, 23]). Increased *MCL-1 *copy number was also more frequently observed in invasive ductal carcinoma compared with normal tissues (TCGA, Oncomine [24]; Additional file 2: Figure S1A). *MCL-1 *mRNA expression correlated with advancing disease stage in the METABRIC dataset (Additional file 2: Figure S1B). Using publically available data sets obtained from NCBI GEO (KM Plotter [25]), we then investigated whether *MCL-1* expression correlates with overall outcome. Three independent *MCL-1* mRNA probes corresponding to Variant 1 (200798_x_at) and full-length *MCL-1* (214057_at and 214056_at) were investigated, and the breast cancer cohort was split into untreated and treated cases (Additional file 2: Figure S1C, D). High *MCL-1* mRNA expression predicted poor outcome in the untreated cases and the effects were reversed in the treated patients (Additional file 2: Figure S1C, D), but the hazard ratios in all cases were modest. Basal and HER2 amplified cases showed poor outcome when *MCL-1 *expression was low, with much stronger hazard ratios of 0.3 (*n* = 94 cases) and 0.08 (*n* = 30 cases) respectively. A similar nonsignificant trend was observed in the luminal A breast cancers (*n* = 68) but not in the luminal B subtypes (*n* = 115).

We then explored the prognostic significance of MCL-1 using tissue microarrays from a cohort of 246 patients diagnosed with invasive breast carcinoma [21]. MCL-1 immunohistochemistry displayed a variable expression pattern across tumors, ranging from absent to strong expression (0–3+) (Fig. 1b). MCL-1 expression was detected in the cytoplasm and in the nucleus of invasive carcinoma cells, with some heterogeneity observed within tumors (Fig. 1c, d). Cytoplasmic MCL-1 levels were lower in HER2-positive tumors compared with luminal A tumors but no other significant differences were observed (Fig. 1c, d). High cytoplasmic MCL-1 levels (Histoscore > 100) were modestly associated with improved breast cancer specific survival (log-rank Mantle Cox *p* = 0.04, hazard ratio = 0.54, 95% confidence interval 0.295–0.975; Fig. 1e). Nuclear MCL-1 expression was not predictive of outcome and no other significant effects on survival were observed when the cases were split into subtypes. High cytoplasmic MCL-1 expression was also positively correlated with the expression of the pro-apoptotic protein PUMA (chi-squared *p* < 0.0001, relative risk 1.75, 95% confidence interval 1.302–2.338).

### Models of MCL-1 antagonism using BIMs2A, a MCL-1 specific sequence

To investigate whether MCL-1 antagonism was sufficient to induce cell death, genetic models were generated using the MCL-1 posit﻿ive breast cancer cell lines MDA-MB-468 and MDA-MB-231. These lines are representative of the triple-negative subtype, which is often the most difficult to treat due to rapid disease progression [26]. The MDA-MB-468 cell line is characteristic of Basal A tumors (Fig. 1a indicated in red) while the MDA-MB-231 cell line is typical of claudin-low tumors (Fig. 1a indicated in blue). MCL-1 protein is found in both cell lines with lower levels observed in the MDA-MB-231 cell line.

**Fig. 2:**
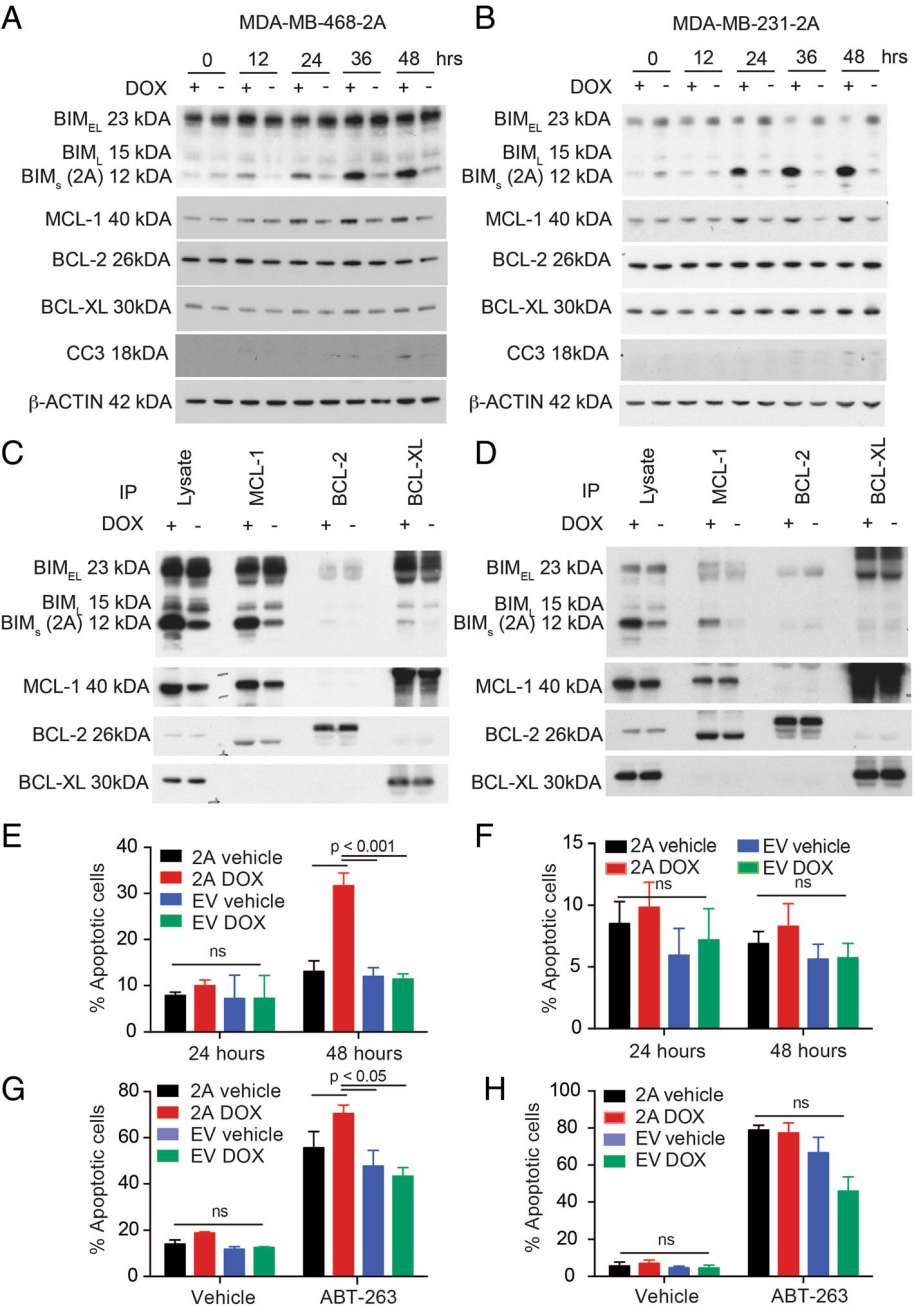
MCL-1 antagonism induced cell death in MDA-MB-468-2A cells but not MDA-MB-231-2A cells grown as monolayers on plastic. Representative western blots of BIM, MCL-1, BCL-2, BCL-XL and Cleaved Caspase 3 (*CC3*) in (**a**) MDA-MB-468-2A and (**b**) MDA-MB-231-2A cells at 0, 12, 24, 36 and 48 hours after treatment with DOX or vehicle. Representative western blots showing the levels of BIM, MCL-1, BCL-2, and BCL-XL following immunoprecipitation with antibodies to MCL-1, BCL-2 and BCL-XL in (**c**) MDA-MB-468-2A and (**d**) MDA-MB-231-2A cells 24 hours after treatment with DOX or vehicle. Bar graphs depicting the average fraction of apoptotic cells (Annexin V-positive) in (**e**) MDA-MB-468-2A or empty vector (*EV*) and (**f**) MDA-MB-231-2A or EV cells treated with DOX or vehicle at 24 and 48 hours. (**g**) Bar graphs depicting the average fraction of apoptotic cells in MDA-MB-468-2A or MDA-MB-468-EV and (**h**) MDA-MB-231-2A or MDA-MB-231-EV cells treated with DOX or vehicle and treated with ABT-263 or vehicle at 24 hours. All graphs and western blots are the average of three independent experiments. *Bars* indicate significantly significant groups, *p* value paired *t* tests. *DOX* doxycycline, *MCL-1* myeloid cell leukemia 1 (Color figure online)

To antagonize MCL-1, we expressed the *BIMs2A* sequence, a BIMs mutant (L62A/F69A) that selectively binds MCL-1 over BCL-2, BCL-XL, BCL-W and BFL-1 [15], using a DOX-inducible expression system. BIMs2A mimics small molecule BH3 mimetics by binding to the hydrophobic pocket and stabilizing MCL-1 levels. DOX treatment strongly induced BIMs2A by 24 hours in both MDA-MB-468 (MDA-MB-468-2A) and MDA-MB-231 (MDA-MB-231-2A) cell lines (Fig. 2a, b). Increased MCL-1 was observed in both cell lines with no alteration in the levels of BCL-2 or BCL-XL (Fig. 2a, b), consistent with the effects observed in mouse embryonic fibroblasts [15]. MCL-1, BCL-XL and BCL-2 immune precipitates showed that endogenous MCL-1:BIM, BCL-2:BIM and BCL-XL:BIM complexes were present in both cell lines in control conditions (Fig. 2c, d). Induction of BIMs2A resulted in BIMs2A binding preferentially to MCL-1 as expected (Fig. 2c, d).

### MCL-1 antagonism induced cell death of MDA-MB-468-2A cells and suppressed invasion of MDA-MB-231-2A cells in a 3D organotypic collagen I/fibroblast invasion assay

MCL-1 antagonism induced apoptosis of MDA-MB-468-2A cells grown as monolayers on plastic, seen as increased caspase 3 cleavage (Fig. 2a) and Annexin V/PI positivity (Fig. 2e), 48 hours after induction of BIMs2A. There was no effect observed in EV control cells treated with DOX (Fig. 2e). No significant death was observed in MDA-MB-231-2A cells grown on plastic (Fig. 2f). MDA-MB-468-2A cells were slightly more sensitive to the BCL-2/BCL-XL inhibitor ABT-263 after 24 hours of DOX exposure (Fig. 2g), suggesting that BCL-2/BCL-XL conferred partial resistance to MCL-1 inhibition and/or that MCL-1 antagonism sensitized the cells to ABT-263. MDA-MB-231-2A cells were highly sensitive to ABT-263 as described previously [14], but no additional effects were observed when combined with MCL-1 inhibition (Fig. 2h).

*MCL-1* knockdown was compared with induction of BIMs2A in all cell lines (Additional file 3: Figure S2A). Cultures of MDA-MB-468-2A and MDA-MB-468-EV cells were mostly destroyed by MCL-1 knockdown via siRNAs targeting *MCL-1*, with significant effects of MOCK or nontargeting transfection alone (Additional file 3: Figure S2A, upper panels). This observation illustrates an artifact produced by knocking down an apoptosis suppressor using reagents that induce apoptosis. MDA-MB-231-2A or MDA-MB-231-EV cells, which have low levels of MCL-1, were insensitive to knockdown of MCL-1, as they were to BIMs2A. These data support our findings using the BIMs2A antagonist, and show the benefit of the mimetic approach over knockdown.

We then examined the effects of small molecule inhibitors of MCL-1, A1210477 [4] and UMI-77 [27], on apoptosis in these 2D models (Additional file 3: Figure S2B). Treatment with 5 μM A1210477 or UMI-77 was as effective as BIMs2A in MDA-MB-468-2A cells, with greater effects observed at 10 μM. Once again MDA-MB-231-2A cells were largely insensitive. A1210477 and UMI-77 also produced a significant increase in death in MDA-MB-157 cells, which also have relatively high levels of MCL-1, but no significant effects of these agents were observed in the MCL-1 low HCC1937 cells. Hence, the current pharmaceutical inhibitors of MCL-1 mimic induction of BIMs2A.

**Fig. 3:**
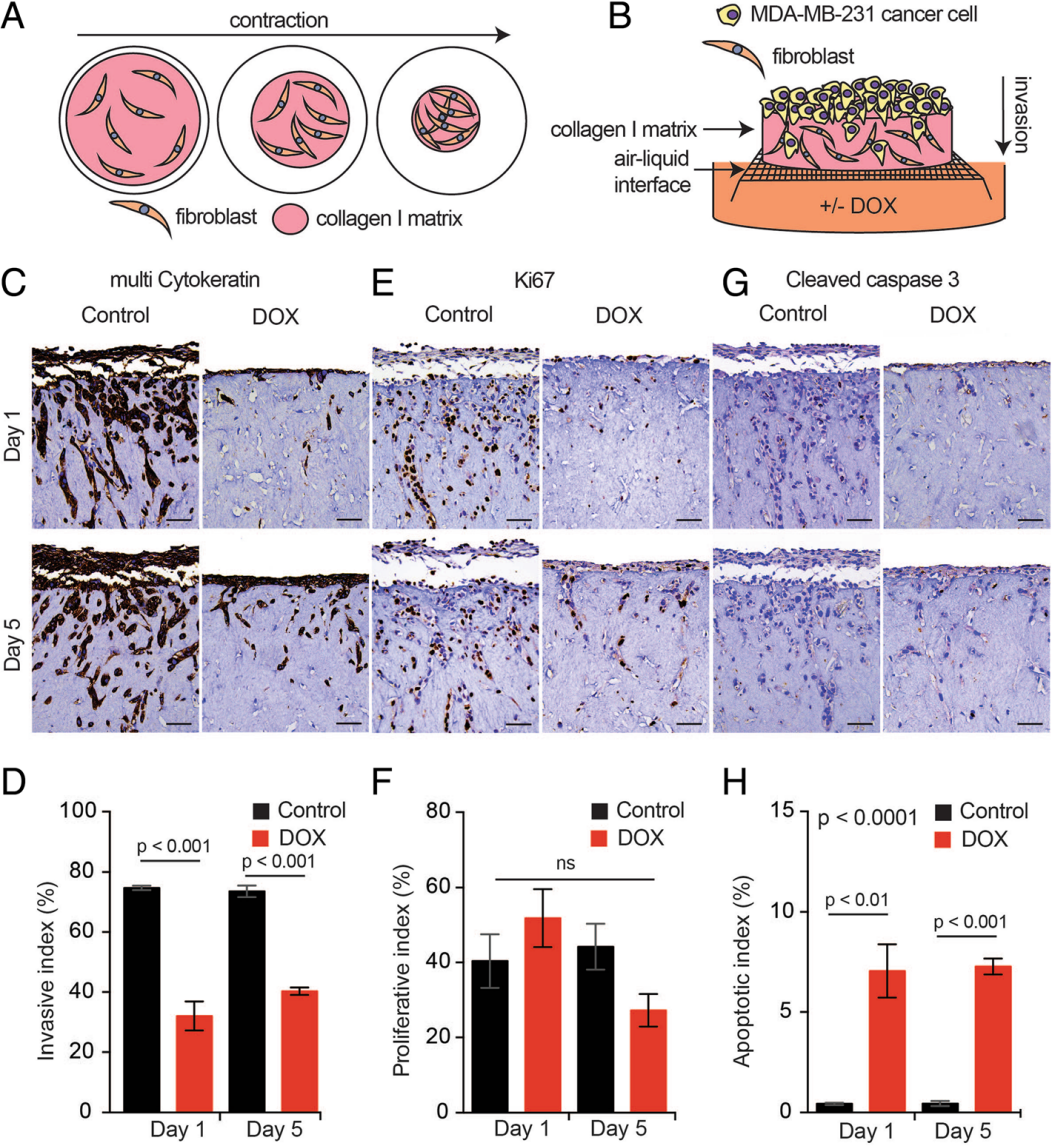
MCL-1 antagonism suppressed invasion and induced apoptosis of MDA-MB-231-2A cells in a 3D organotypic invasion assay. (**a**) Schematic of collagen I/fibroblast matrix contraction and (**b**) invasion assay with MDA-MB-231-2A cells invading through a contacted matrix towards a chemo-attractive gradient created by an air–liquid interface. Representative immunohistochemistry images of antibodies to multi-cytokeratin (**c**), Ki67 (**e**) and Cleaved Caspase 3 (**g**) on contracted matrices seeded with MDA-MB-231-2A cells treated with DOX or vehicle (*Control*) at day 1 and day 5 of invasion. Bar graphs depicting the average invasive index (**d**; total number of cells within the matrix expressed as a proportion of the number of cells on top with the opportunity to invade), the average proliferative index (**f**; ratio of Ki67-positive cells to total number of cells) and the average apoptotic index (**h**; ratio of Cleaved Caspase 3-positive cells to total number of cells). Average of three independent experiments. *Bars* indicate significantly significant groups, *p* value unpaired *t* tests. *DOX* doxycycline (Color figure online)

The effects of MCL-1 antagonism were also examined in a 3D organotypic in vitro model of invasion that more accurately recapitulates key aspects of the in vivo microenvironment [20]. MDA-MB-231-2A cells were seeded onto contracted collagen I matrices embedded with fibroblasts (Fig. 3a), transferred to an air–liquid interface and allowed to invade within the chemo-attractive gradient over a period of 10 days (Fig. 3b). BIMs2A was induced with supplementation of DOX in the media on day 1 of invasion (Fig. 3c–h, day 1). DOX treatment resulted in a significant reduction in the ability of MDA-MB-231-2A cells to invade (Fig. 3c, d), independent of potential effects on proliferation (Fig. 3e, f). In a post-invasion scenario, where we allowed the cells to invade for 5 days before DOX treatment (Fig. 3c–h, day 5), invasion was also significantly reduced (Fig. 3c, d) and proliferation was unaffected (Fig. 3e, f). As expected, induction of BIMs2A resulted in a modest increase in apoptosis in both conditions; however, apoptotic cells accounted for only a small proportion of the total population of cells and therefore could not account for the observed large reduction in invasion (Fig. 3g, h). MCL-1 antagonism prior to or after the initiation of invasion thus has anti-invasive effects in vitro.

### MCL-1 antagonism impaired tumor growth and reduced lung metastasis

**Fig. 4:**
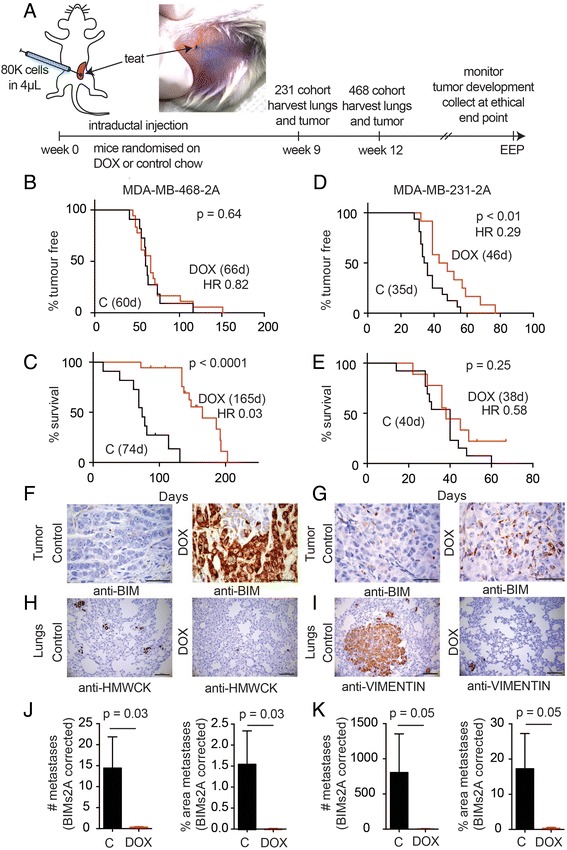
MCL-1 antagonism delayed tumor growth and suppressed metastasis in triple-negative breast cancer xenografts. (**a**) Schematic of the intraductal xenograft models and photograph of an intraductal xenograft immediately after injection of 80,000 breast cancer cells in 4 μl 0.1% Trypan blue/2% FCS in PBS. Kaplan–Meier survival curves of (**b**, **d**) time to detection and (**c**, **e**) time from detection to ethical end point (EEP) of mice bearing MDA-MB-468-2A (**b**, **c**) or MDA-MB-231-2A (**d**, **e**) xenografts fed DOX or control food. (# **d**) Median days. Log-rank *p* values. Immunohistochemistry using an antibody raised against human BIM showing the expression of BIMs2A in the tumors of mice bearing (**f**) MDA-MB-468-2A or (**g**) MDA-MB-231-2A xenografts fed DOX or control food (*scale bars* 50 μm). Immunohistochemistry using (**h**) an antibody to human high molecular weigh cytokeratin (*HMWCK*) in the lungs of mice bearing MDA-MB-468-2A xenografts and (**i**) human Vimentin in the lungs of mice bearing MDA-MB-231-2A xenografts fed DOX or control food (*scale bars* 100 μm). Quantitation of the average number (*#*) and % area of lung metastasis corrected for the levels of BIMs2A detected in the tumors of mice bearing (**j**) MDA-MB-468-2A xenografts and (**k**) MDA-MB-231-2A xenografts after mice were fed DOX or control food. All graphs are representative of three independent experiments. *Bars* indicate significantly significant groups, *p* value unpaired *t* tests. *DOX* doxycycline, *HR* hazard ratio (Color figure online)

The effects of MCL-1 antagonism on invasion were examined in vivo using a mammary intraductal injection technique [18], which requires cells to breach the basement membrane of the mammary duct to become locally invasive or metastatic (Fig. 4a)*.* Mammary intraductal injection places breast cancer cells in a mammary tissue niche during tumor establishment and this maintains the phenotypic characteristics of the xenograft [28]. We observed that intraductal injection promotes metastatic progression in MDA-MB-468-2A cells. In mice bearing xenografts of MDA-MB-468-2A cells, induction of BIMs2A significantly reduced the rate of tumor growth, doubling the survival times of control mice (median survival: DOX 165 days vs Control 74 days; Fig. 4b, c and Additional file 3: Figure S2A, B). Induction of BIMs2A reduced the rate of onset of MDA-MB-231-2A xenografts (Fig. 4d), but there was no significant effect of BIMs2A on subsequent time to ethical end point (median survival: DOX 38 days vs Control 40 days; Fig. 4e). Immunohistochemistry confirmed strong and heterogeneous expression of BIMs2A in MDA-MB-468-2A primary tumors (Fig. 4f, DOX) and MDA-MB-231-2A primary tumors (Fig. 4g, DOX), confirming that BIMs2A was induced in the tumors.

We next investigated the effects of MCL-1 antagonism on the ability of triple-negative breast cancer cells to disseminate to the lung, using cohorts of mice bearing MDA-MB-468-2A and MDA-MB-231-2A mammary intraductal xenografts. Tumors and lungs were collected from these mice at 9 (MDA-MB-231-2A) and 12 weeks (MDA-MB-468-2A) (Fig. 4a), and lung metastases including single cell foci were visualized using anti-human pan-Cytokeratin or Vimentin immunohistochemistry (Fig. 4h and i, respectively). BIMs2A levels in tumors and lungs was analyzed using anti-human BIM immunohistochemistry (Additional file 4: Figure S4A and B respectively). A range of BIMs2A expression was observed among the individual cells of the primary tumors produced from MDA-MB-468-2A and MDA-MB-213-2A cell lines in mice fed DOX food (Additional file 4: Figure S4A). This observation was expected because the xenografted material was derived from pools of SH570MK-BIMs2A-infected cells. There were fewer and smaller metastases in the lungs from DOX-fed mice (Additional file 4: Figure S4C, D), however, these metastases lacked BIMs2A expression despite a predominance of cells expressing BIMs2A in the primary tumors (Additional file 4: Figure S4B), showing that only cells which did not express the BIMs2A antagonist could metastasize. To statistically analyze this effect we corrected these data using levels of BIMs2A expression observed in primary tumors (Additional file 4: Figure S4A, quantified in Additional file 4: Figure S4E, F), and showed a significant reduction in both the size and number of metastases in mice bearing both MDA-MB-468-2A and MDA-MB-231-2A xenografts (Fig. 4j, k and Additional file 4: Figure S4G, H). These data show, through a cellular competition assay, that MCL-1 antagonism by BIMs2A potently suppresses the ability of a tumor cell to metastasize.

We investigated whether MCL-1 antagonism could suppress cell seeding to the lung via tail vein injection (Additional file 4: Figure S4I–L)*.* In this model, MCL-1 antagonism produced a trend towards reduced number and area of lung metastases produced from tail vein injections of MDA-MB-468-2A (Additional file 4: Figure S4I), but significantly suppressed the ability of MDA-MB-231-2A cells to survive and colonize the lung (Additional file 4: Figure S4J), where we observed an almost complete reduction in the size and number of lung metastases. Immunohistochemistry once again revealed that all cells that had colonized the lungs did not express the BIMs2A antagonist (Additional file 4: Figure S4K, L), indicating (as observed in the earlier intraductal models of metastasis) that only those cells which did not express the BIMs2A antagonist were able to survive and colonize the lung. Hence, expression of the BIMs2A ligand inhibited lung colonization. In addition we treated a cohort of mice injected with MDA-MB-157 cells with A1210477 or vehicle and observed a trend toward reduced metastases size that did not reach statistical significance (Additional file 4: Figure S4K). Importantly these mice showed no overt signs of systemic toxicity, suggesting that a therapeutic window for systemic MCL-1 suppression can be achieved. Together our data show that MCL-1 antagonism suppresses metastatic progression.

### MCL-1 antagonism increased the sensitivity of MDA-MB-231-2A cells to dasatinib

We then explored how MCL-1 might be regulating metastatic progression by profiling the kinome of MDA-MB-468-2A cells 24 hours after BIMs2A induction, prior to the onset of apoptosis, using Kinexus Antibody Microarrays. MCL-1 antagonism altered the levels of a large number of proteins important for invasion (Additional file 5: Figure S5A) [29, 30]. We observed changes in many SRC family kinases and their targets important in invasion, including a small increase in total CSK (cSRC tyrosine kinase), a negative regulator of SRC family kinases [31] (gain +53%). We also observed decreased total levels of the SRC family kinase, FYN, and the SFK target, ABL, which was increased by MCL-1 antagonism (63% and –20%, respectively). Loss of phosphorylation was also detected for Paxillin (–63%) and Vimentin (–71%), targets of SRC family kinase activity [32, 33]. MCL-1 antagonism also decreased the auto-phosphorylation site Y1148 in EGFR (–67%), proposed to be involved in the regulation of invasion by Src [34]. These data suggest that MCL-1 antagonism modulated the output of the SRC family kinases.

We also detected an increase in S3 phosphorylation of Cofilins 1 and 2 (+31% and +138%, respectively), an additional protein important for invasion. Cofilin is localized to invadopodia in MDA-MB-231-2A cells where phosphorylation of serine 3 suppresses actin-severing and polymerization function [29, 30]. Western blot analysis revealed that the ratio of S3 Cofilin to total Cofilin was significantly greater in MDA-MB-231-2A intraductal xenografts treated with DOX compared with control, with a similar trend observed in MDA-MB-468-2A xenografts (Additional file 5: Figure S5C). Immunofluorescence using antibodies against total Cofilin and phosphorylated Cofilin in MDA-MB-231-2A cells grown as monolayers also showed the same effect, with DOX treatment resulting in lower total Cofilin levels and increased serine 3 phosphorylation (Additional file 5: Figure S5D). Finally, proximity ligation assays using specific antibodies to MCL-1 and Cofilin in MDA-MB-231-2A cells revealed a novel interaction of MCL-1 with Cofilin that occurs throughout the content of MDA-MB-231-2A cells (Additional file 5: Figure S5E). Hence our data suggest that MCL-1 may regulate two modes of invasion: firstly those regulated by the SRC family kinases, and secondly those that involve the cytoskeletal remodeling protein Cofilin.

**Fig. 5:**
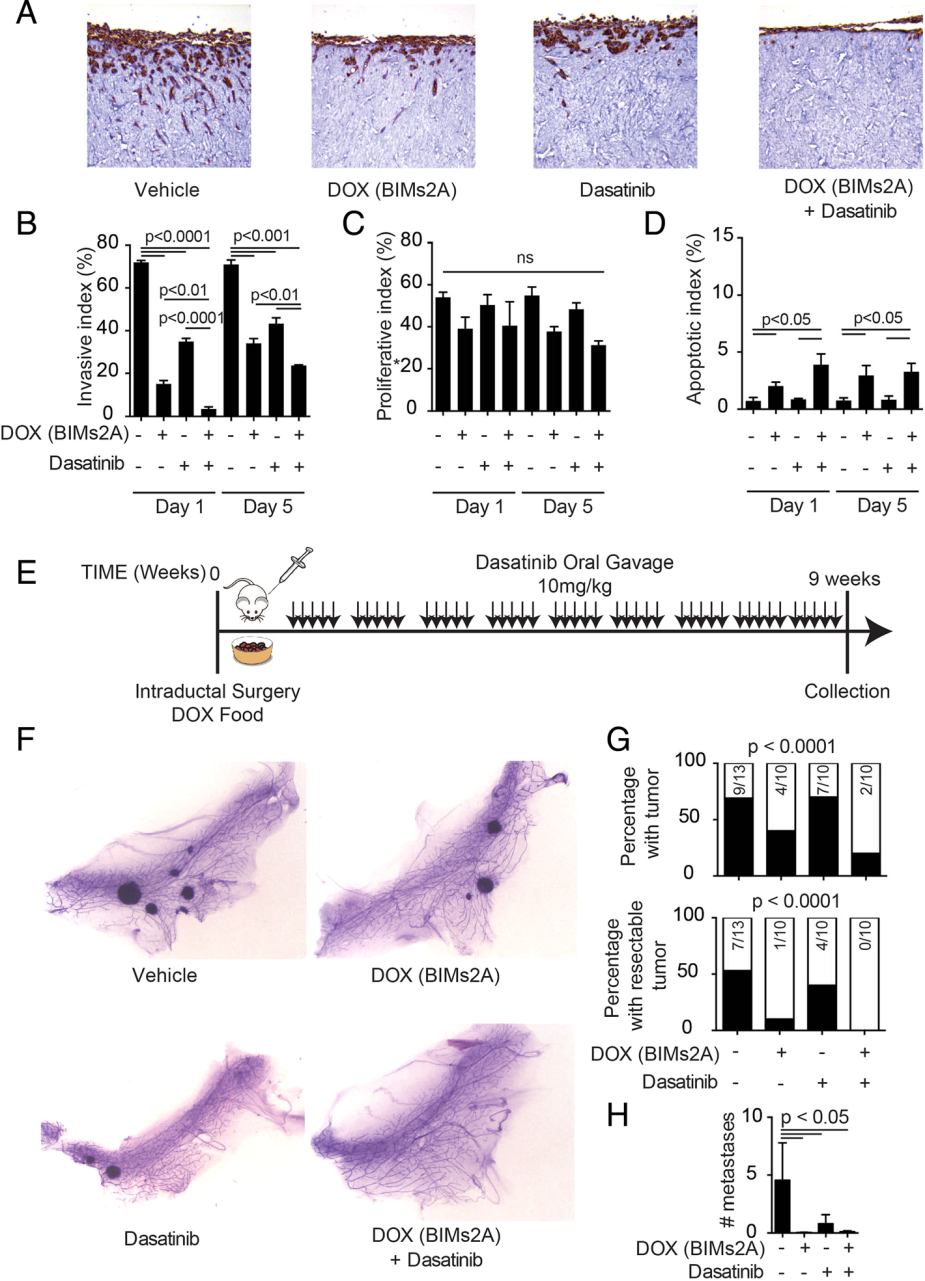
MCL-1 antagonism increased the sensitivity of MDA-MB-231-2A cells to dasatinib. (**a**) Representative immunohistochemistry images using antibodies against multi-Cytokeratin on contracted matrices seeded with MDA-MB-231-2A cells treated with vehicle, DOX, dasatinib or their combination at day 1 invasion and collected 10 days post invasion. Bar graphs depicting the average invasive index (**b**; total number of cells within the matrix expressed as a proportion of the number of cells on top with the opportunity to invade), the average proliferative index (**c**; ratio of Ki67-positive cells to total number of cells) and the average apoptotic index (**d**; ratio of CC3-positive cells to total number of cells). Average of three independent experiments. *Bars* indicate significantly significant groups, *p* value unpaired *t* tests. (**e**) Schematic representation of BIMs2A and dasatinib combination experiment using MDA-MB-231-2A in vivo mammary intraductal xenografts. (**f**) Representative Carmine-stained mammary whole mounts of MDA-MB-231-2A mammary intraductal xenografts 9 weeks after inoculation from mice treated with vehicle (*n* = 13), DOX (*n* = 10), dasatinib (*n* = 10) and a combination of both (*n* = 10). (**g**) Bar graphs depicting the total number of tumors (resectable and present in the mammary whole mount) or palpable from mice with MDA-MB-231-2A mammary intraductal xenografts 9 weeks after inoculation. Chi-squared *p* value and numbers of mice indicated within the bars. (**h**) Number of metastases from mice treated with vehicle, DOX, dasatinib and a combination of both. *Bars* indicate statistically significant groups, Mann–Whitney *p* value. *DOX* doxycycline

Because there are pharmaceutical compounds that target SRC family kinases, such as dasatinib, which have been shown to be anti-invasive in multiple cancer types [35–37], we assessed the efficacy of combining MCL-1 antagonism with dasatinib treatment. When grown as monolayers on plastic, MDA-MB-468-2A cells were sensitive to dasatinib as a single agent alone, but DOX induction of BIMs2A amplified these effects (Additional file 6: Figure S6, top left panel). MDA-MB-468-EV control cells were equally sensitive to dasatinib and DOX treatment had no further effect (Additional file 6: Figure S6, lower left panel). MDA-MB-231-2A and MDA-MB-231-EV cells were largely insensitive to dasatinib as a single agent when grown as monolayers in 2D (Additional file 6: Figure S6, right panels). In contrast, when seeded onto contracted collagen I matrices, dasatinib significantly reduced their invasive capacity (Fig. 5a, b), and induction of BIMs2A significantly enhanced this effect, particularly when treatment was administered at day 1 of matrix seeding (Fig. 5a, b). BIMs2A, dasatinib or their combination had no effect on cell proliferation over the 10-day course of the experiment, whether treated at seeding or 5 days of invasion (Fig. 5a, c). Dasatinib also had no effects on apoptosis in MDA-MB-231-2A cells as a single agent (Fig. 5a, d). BIMs2A produced a small but significant increase in apoptosis in these cells when treatment was initiated at seeding or after 5 days of invasion, and dual therapy increased these effects (Fig. 5a, d).

In MDA-MB-231-2A intraductal xenografts in vivo, single-agent dasatinib had limited effects. Induction of MCL-1 inhibition with DOX alone had a slightly greater effect than dasatinib alone. Treatment with DOX and dasatinib significantly delayed the onset of mammary tumorigenesis (Fig. 5f, g), and metastasis to the lung was inhibited in all conditions compared with control (Fig. 5h).

## Discussion

MCL-1 amplification occurs in approximately 30% of breast cancers and increased expression is associated with the acquisition of chemo-resistance and relapse [8], making MCL-1 an attractive therapeutic target. In our study, cytoplasmic MCL-1 protein was detected in all clinico-pathological subtypes of invasive breast cancer and breast cancer cell lines. High cytoplasmic MCL-1 protein levels correlated with high expression of PUMA. MCL-1 has previously been shown to be coexpressed with BIM across all subtypes of invasive breast carcinoma [14] and this expression pattern was recapitulated in our panel of human breast cancer cell lines. Endogenous MCL-1:BIM complexes are also present in the responsive triple-negative breast cancer cell lines in this study. Potentially these complexes might reveal tumors that are ready to respond to therapy [38]. This hypothesis is supported by the data presented from published data sets and tissue microarrays showing that the highest levels of *MCL-1 *mRNA and protein were protective for overall survival in treated cases. MCL-1 levels in triple-negative and HER2 breast cancers may provide a biomarker of response to routine chemotherapy (basal cancers) and anti-HER2 therapy (HER2 amplified cases). Importantly, the data presented reveal that for tumors with the poorest outcome, which have the lowest levels of MCL-1 (i.e., represented by the MDA-MB-231 cell line), MCL-1 antagonism via a BH3-mimetic strategy can inhibit invasion and metastatic progression and is effective in combination with SRC family kinase inhibition by dasatinib.

Inducible expression of the specific MCL-1 inhibitory sequence BIMs2A produced disparate responses in MDA-MB-468-2A and MDA-MB-231-2A cell lines. BIMs2A expression resulted in apoptosis of MCL-1 high MDA-MB-468-2A cells in 2D cultures and more than doubled the median survival times of mice bearing MDA-MB-468-2A xenografts. In contrast, MCL-1 antagonism had limited effects on MCL-1 low MDA-MB-231-2A cells when grown as monolayers in vitro but delayed tumor onset in mice bearing MDA-MB-231-2A xenografts*.* One reason for this discrepancy may be the differential expression of MCL-1 in both cell lines. In support of this hypothesis, we showed that MDA-MB-157 cells, which also have high levels of MCL-1, were sensitive to pharmacological inhibition of MCL-1, but that this was not apparent in the MCL-1 low HCC-1937 cells. Also, MDA-MB-231-2A cells are dependent on BCL-2/XL for their survival, as indicated by their sensitivity to ABT-263. Further, the levels of the MCL-1 interacting partners (e.g., pro-apoptotic BH3-only proteins) may play a role in sensitivity to MCL-1 antagonism as has been reported before [39]. For example, MDA-MB-231 cell lines have lower BIM levels than the other three cell lines examined in this study. Another possibility is context specificity. MCL-1 antagonism produced a small but significant effect on apoptosis in MDA-MB-231-2A cells in the 3D organotypic model system, increased the latency to the onset of tumorigenesis and suppressed metastasis in vivo. Hence signaling from the extracellular matrix may play an important role in sensitivity to MCL-1 antagonism. Interestingly, both cell lines are sensitive to anoikis when assayed in PolyHEMA assays, although BIMs2A increased this effect only in MDA-MB-468-2A cell lines (Additional file 7: Figure S7). Finally, xenografted MDA-MB-468-2A tumors grew more slowly than MDA-MB-231-2A tumors (Additional file 8: Figure S3), perhaps accounting for the difference in tumor end point specific survival.

Metastasis requires directed cellular invasion into the host stroma and is stimulated by chemo-attractants, and requires remodeling of the cytoskeleton, dynamic membrane changes, cellular invasion and localized destruction of the host stroma [40]. Using 3D organotypic cultures in vitro and mammary intraductal xenografts in vivo, which more faithfully recapitulate this important tissue context, we revealed a previously undiscovered function of MCL-1. MCL-1 antagonism profoundly suppressed invasion and metastasis in our 3D organotypic model and in xeno﻿grafts in mice. The weakly metastatic MDA-MB-468 and MDA-MB-157 cells could not be used in the 3D organotypic model in vitro, but showed similar effects on metastatic progression in response to MCL-1 antagonism in vivo. Kinomic profiling suggested that two key pathways involved in invasion were altered in response to MCL-1 antagonism. The first pathway via modulation of the activity of SRC family kinases, important proteins involved in invasion, and the second via a potential interaction with Cofilin [30, 41]. Although currently beyond the scope of this manuscript, future work understanding the invasion networks regulated by MCL-1 will yield insights into novel targeted approaches that inhibit metastasis. Importantly, the data presented show that in addition to its roles in survival, mitochondrial function and cell cycle, MCL-1 also regulates invasion.

We also showed that dual targeting of MCL-1 by BIMs2A and SRC family kinases by dasatinib potently suppressed invasion and the onset to tumorigenesis. Phase II clinical trials of single-agent dasatinib have shown durable and objective clinical responses in a small proportion (5%) of patients with locally advanced and metastatic triple-negative breast cancer [42]. Combination trials show better results; for example, phase I clinical trials of dasatinib with capecitabine show clinical response rates of 56% in unselected patients. Other trials combining dasatinib with paclitaxel and bosutinib with exemestane are currently underway in patients with advanced metastatic breast cancer and are reporting improved responses compared with single agents alone. Hence although some promising results have been observed for SRC family kinase inhibitors, like dasatinib, in clinical trials for breast cancer, the efficacy of this and other SRC family kinase inhibitors will likely be improved by combining these drugs with others that increase potency. The data presented here suggest that MCL-1 antagonism via small molecule BH3 mimetics may provide a potential way to enhance the effects of SRC family kinase inhibitors.

## Conclusions

The data presented provide first evidence for the efficacy of MCL-1 inhibition for the suppression of metastasis in triple-negative breast cancer. We revealed a previously undiscovered role for MCL-1 as a regulator of invasion, and thus MCL-1 does not merely act as a regulator of survival. This study provides the first in-vivo evidence to support the efficacy of MCL-1 BH3 mimetics [4, 43] in triple-negative breast cancer as single agents, and in combination with SRC family kinase inhibitors such as dasatinib.

## Additional files

Additional file 1: showing supplementary materials and methods. (DOCX 31 kb)

Additional file 2: Figure S1. showing MCL-1 expression in human breast cancer. (**A**) Box–whisker plot depicting the average copy number units (log_2_) of MCL-1 in invasive ductal breast carcinoma (*IDC*) in the TCGA2012 cohort: normal *n* = 111, IDC *n* = 638. Median, and first and third quartiles indicated by the *box*, data minimum and maximum points by the *bars*. (**B**) Box–whisker plot, format as for (**A**), depicting the mean centered intensity of MCL-1 mRNA expression in invasive breast carcinoma across stages in the METABRIC dataset. Stage 0 *n* = 425, Stage I *n* = 257, Stage I *n* = 446, Stage II *n* = 69 and Stage IV *n* = 8. (**C**) Kaplan–Meier survival curves and log-rank *p* values of time to overall survival of patients with triple-negative breast cancer and with MCL-1 CNV alteration. Kaplan–Meier survival curves of overall survival using cases from KM Plotter time using three independent *MCL-1* mRNA probes corresponding to Variant 1 (200798_x_at) and full-length *MCL-1* (214057_at and 214056_at). Separate Kaplan–Meier survival curves are provided for untreated and treated cases. Treated cases were then split into individual curves split by histopathological subtype. Significant log-rank *p* values are proved in *red* and nonsignificant values in *black* (**D**) Table depicting the number of cases and the range and optimal cutoff probe values used for each of the Kaplan–Meier curves from (**C**). (JPG 2111 kb)

Additional file 3: Figure S2. showing the apoptotic effect of *MCL-1* siRNA and MCL-1 inhibitors on MDA-MB-468-2A and MDA-MB-231-2A, MDA-MB-157 and HCC-1937 cells. (**A**) *MCL-1* siRNA. Bar graphs depicting the average fraction of apoptotic cells, measured as total Annexin V-positive by flow cytometry, for cells and treatments as indicated at 48 hours. *Error bars* show standard error of the mean. (**B**) MCL-1 inhibitors. Bar graphs of apoptosis as measured in (**A**) for cells and treatments as indicated. For A1210477 and UMI-77: + treated with 5 μM, ++ treated with 10 μM. All graphs are the average of three independent experiments. *Bars* indicate statistically significant groups, *p* value unpaired *t* tests. (JPG 1192 kb)

Additional file 4: Figure S4. showing BIMs2A expression is induced by DOX in MDA-MB-468-2A and MDA-MB-231-2A xenograft tumors but not induced in the cells from these xenografts that formed the lung metastases. Representative immunohistochemistry images using an antibody to human BIM in the tumors (**A**) and the lungs (**B**) of mice bearing MDA-MB-468-2A and MDA-MB-231-2A intraductal xenografts fed DOX or control food. *Dotted lines*, areas of metastases. Quantitation of the average raw numbers (*#*) and % area of metastasis in the lungs of mice bearing (**C**) MDA-MB-468-2A (*n* = 7) and (**D**) MDA-MB-231-2A (*n* = 6) intraductal xenografts after mice were fed DOX or control (**C**) food. Comparison of the average intensity of human BIM (BIMs2A) in the tumors and the corresponding lung metastases of mice bearing (**E**) MDA-MB-468-2A (*n* = 7) and (**F**) MDA-MB-231-2A (*n* = 6) intraductal xenografts after mice were fed DOX or control (**C**) food. Expression of lung metastatic burden relative to the levels of BIMs2A expression in tumors of (**G**) MDA-MB-468-2A (*n* = 7) and (**H**) MDA-MB-231-2A (*n* = 6) intraductal xenografts. Quantitation of the average number (*#*) of metastasis and % area of metastasis in the lungs of mice given tail vein injections of (**I**) MDA-MB-468-2A (*n* = 10) and (**J**) MDA-MB-231-2A (*n* = 10) and given DOX or control (**C**) food. Quantitation of the average intensity of human BIM (BIMs2A) in the lungs of mice given tail vein injections of (**K**) MDA-MB-468-2A (*n* = 10) and (**L**) MDA-MB-231-2A 2A (*n* = 10) and given DOX or control (**C**) food. Quantitation of the average number (*#*) of metastasis and % area of metastasis in the lungs of mice given tail vein injections of (**M**) MDA-MB-157 cells and treatment with A1210477 (*n* = 5) or vehicle control (*n* = 5). *Bars* indicate statistically significant groups, Mann–Whitney *p* value. (JPG 2091 kb)

Additional file 5: Figure S5. showing that MCL-1 antagonism resulted in changes in proteins involved in SRC family kinase signaling and phosphorylation at serine3 of Cofilin*.* (**A**) Normalized *z*-ratio (a measure of statistical significance) of phosphorylated and total proteins (as indicated) in MDA-MB-468-2A cells at 24 hours after treatment with DOX compared with control cells. (**B**) Western blots of serine 3 phosphorylated Cofilin, total Cofilin and Actin in xenografts of MDA-MB-468-2A and MDA-MB-231-2A fed DOX food as indicated. (**C**) Bar graphs depicting the ratio of serine 3 phosphorylated Cofilin to total Cofilin from (**B**). *Bars* indicate statistically significant groups, Mann–Whitney *p* value. (**D**) Immunofluorescence of Cofilin and p-Cofilin MDA-MB-231-2A cells grown on fibronectin 24 hours after DOX or vehicle treatment. (**E**) Proximity ligation assays using antibodies to MCL-1 and Cofilin (*green*), Phalloidin (*red*) and Dapi (*blue*) in MDA-MB-231-2A cells. (JPG 1419 kb)

Additional file 6: Figure S6. showing that MCL-1 antagonism and Dasatinib treatment induced apoptosis in MDA-MB-468-2A cells but not MDA-MB-231-2A cells when grown in 2D monolayer cultures. Bar graphs depicting the average fraction of apoptotic cells (total Annexin V-positive by flow cytometry) as indicated after 24 hours after treatment with vehicle, DOX, 5 μM A1210477 and 5 μM UMI-77 alone and in combination with 1 μM dasatinib after 24 hours. All graphs and western blots are the average of three independent experiments. *Bars* indicate statistically significant groups, *p* value paired *t* tests. (JPG 1029 kb)

Additional file 7: Figure S7. showing that MCL-1 antagonism increased sensitivity to anoikis in MDA-MB-468-2A but not MDA-MB-231-2A cells. Bar graphs depicting the average fraction of apoptotic cells (total Annexin V-positive by flow cytometry) in MDA-MB-468-2A (**A**) and MDA-MB-231-2A (**B**) plated as monolayers in culture (normal) or onto PolyHEMA treated plates and harvested at 24 hours after plating. ANOVA *p* value, *dashes* indicate statistically significant groups. *Bars* indicate statistically significant groups, *p* value paired *t* tests. (JPG 409 kb)

Additional file 8: Figure S3. showing that MCL-1 antagonism by BIMs2A slows tumor growth in mice bearing MDA-MB-468-2A xenografts but not MDA-MB-231-2A xenografts. (**A–F**) Line graphs depicting the tumor growth curves of MDA-MB-468-2A xenografts (**A, B**) and MDA-MB-231-2A xenografts (**C, D**) from mice fed with DOX or control food. Linear regression of these curves shown in **B** and **D** respectively. A comparison of the growth rate of tumors in mice bearing MDA-MB-468-2A (*black*) and MDA-MB-231-2A (*red*) xenografts fed with control food (**E, F**). (JPG 1348 kb)

## Abbreviations

2A: BIMs2A L62A/F69A BIM mutant
AC: Doxorubicin (adriamycin), cyclophosphamide
BAK: BCL-2-antagonist/killer 1 (BCL-2-like 7)
BAX: BCL2-associated X protein (BCL-2-like 4)
BCL-2: B-cell CLL/lymphoma 2
BCL-XL: Apoptosis regulator BCL-X (BCL2-Like 1)
BIM: BCL-2 mediator of cell death (BCL2-Like 11)
BIMs2A: L62A/F69A BIM mutant
CDK1: Cyclin-dependent kinase 1
CHK1: Checkpoint kinase 1
CMF: Cyclophosphamide methotrexate fluorouracil
CNV: Copy number variation
DOX: Doxycycline
EEP: Ethical end point
FISH: Fluorescence in-situ hybridization
HREC: Human Research Ethics Committee
MCL-1: Myeloid cell leukemia 1
NOXA: Phorbol-12-myristate-13-acetate-induced protein 1
PUMA: P53 Up-regulated modulator of apoptosis (BBC3)
SDS: Sodium dodecyl sulfate
SPF: Specific pathogen free
TAM: Tamoxifen
TCGA: The Cancer Genome Atlas
