# Supplementary material for: MCL-1 inhibition provides a new way to suppress breast cancer metastasis and increase sensitivity to dasatinib
Source: Breast Cancer Res. 2016 Dec 8;18:125. doi: 10.1186/s13058-016-0781-6 (PMC5146841; doi:10.1186/s13058-016-0781-6)
Supplement: Supplementary file 1 — showing supplementary materials and methods. (DOCX 31 kb) [file 13058_2016_781_MOESM1_ESM.docx]

**Additional file 1**

**Supplementary materials and methods**

**Mice and mammary intraductal xenografts**

Nine to twelve week old NODScidIL2gamma^-/-^ mice were anaesthetized with Isofluorane and hair shaved in the region superior to the right 4^th^ inguinal mammary gland. Ultrafine scissors were used to remove the tip (approximately 0.5 mm) of the right 4^th^ inguinal nipple. 80,000 BIMs2A inducible MDA-MB-468 or MDA-MB-231 breast cancer cells in 4µl of 0.1% Trypan blue in PBS containing 2% FCS were then injected directly and slowly into the primary mammary duct and complete mammary ductal filling observed through the skin. Mice were then randomized onto DOX-containing CHOW or control-treated groups and monitored twice weekly for tumor growth. For dasatinib experiments mice were treated with 10mg/kg Dasatanib (Bristol-Myers Squibb, Princeton, NJ.) in a Citrate buffer or vehicle control by oral gavage daily as indicated in Fig.5E. Once tumors were palpated, the major and minor axis of each tumor was measured twice weekly and tumor volume calculated using the formula (minor diameter (mm)) ^2^ x (major diameter) divided by 2. At the end of the experiment, as indicated in the figures, mice were injected with BrdU 2 hours before being euthanized with CO2 asphyxiation and tumor, lungs and liver were harvested and fixed for 4 hours in 10% buffered formalin at room temperature. Where possible mammary glands were whole mounted and tissues were processed for histology as below.

**Immunohistochemistry**

Mouse mammary glands were harvested and fixed in 4% buffered formalin for 4 hours. Glands were defatted in 3-4 changes of acetone before being dehydrated and stained in Carmine alum as previously described [1]. After fixation, mammary glands, tumors or lungs were then dehydrated in a series of graded alcohols and embedded in Paraffin for sectioning. Sections were either stained with haematoxylin and eosin for routine histochemistry or stained with antibodies to the following antigens using immunohistochemistry.

**Immunofluoresence**

Cells were plated at a density of 5x10^4^ on gelatin coated coverslips for 12 hours prior to fixation with pre-warmed to 37°C 4% PFA in CSK (20mM PIPES, 200mM KCL, 600mM sucrose, 4mM EGTA and 4mM MgCl_2_ in ddH_2_0) for 30mins. Cells were washed twice with PBS for 5mins then permeabilised using 0.1% Triton-x100 for 10mins before washing twice with PBS for 5mins and blocking with 3% BSA in PBS for 30mins. Coverslips were incubated with the appropriate primary antibody in 3% BSA in PBS overnight at 4°C. Coverslips were washed thoroughly in PBS prior to incubation with secondary antibodies, DAPI and phalloidin stains for 2 hours at RT. Once the incubation was completed coverslips were washed 3x in PBS before being coverslipped on superfrost slides using confocal microscope mounting matrix. Coverslips were allowed to settle overnight prior to imaging on a Leica DM5500 immunofluorescence microscope.

**Proximity Ligation Assays**

Cells were plated and treated as for immunofluorescence up to the primary antibody step. Once cells had been incubated overnight with either MCL-1/Cofilin 1, MCL-1/Rabbit IgG/ Mouse IgG1/Cofilin primary antibodies (see table below for dilution and concentrations used) coverslips were washed twice with PBS for 5mins before starting the proximity ligation assay (PLA) using Duolink reagents (Sigma). All PLA steps from PLA probe incubation, ligation and amplification were carried out according to the manufacturers instructions. Coverslips were mounted on superfrost slides using confocal microscope mounting matrix. Coverslips were allowed to settle overnight prior to imaging on a Leica DM5500 immunofluorescence microscope.

| Antigen | Antibody | Species reactivity | Retrieval | Primary antibody conc. | Secondary antibody |
| --- | --- | --- | --- | --- | --- |
| BIM | C34C5 Cell Signaling | Human>Mouse | pH9 S2367 Pressure Cooker 30secs | 1:150 | Envision Rabbit (K4009) |
| High Molecular Weight Cytokeratin | 34ßE12 DAKO | Human | pH9 S2367 Pressure Cooker 30secs | 1:100 | Envision Mouse (K4007) |
| Vimentin | V9 Leica Biosystems | Human | pH9 S2367 Pressure Cooker 30secs | 1:400 | Envision Mouse (K4007) |
| MCL-1 | MA5-13932 ThermoFischer (Pierce) | Human | pH6 S2367 Pressure Cooker 4mins | 1:20NoHi | Envision Mouse (K4007) |
| Cleaved Caspase 3 | Asp175 5A Cell Signaling | Mouse/ human | pH9 S2367 Pressure Cooker 30secs | 1:100 | Envision Rabbit (K4009) |
| BrdU | Bu20a M0744 Dako | mouse | pH9 S2367 Waterbath 20 mins | 1:100 | Envision Mouse (K4007) |
| Ki67 | SP6 ThermoScientific | human | pH9 S2367 Pressure Cooker 30secs | 1:500 | Envision Mouse (K4007) |
| Multi- Cytokeratin | C-11 Leica-Novocastra | human | pH9 S2367 Pressure Cooker 30secs | 1:50 | Envision Mouse (K4007) |
| Total Cofilin 1 | 5175 (D3F9 clone) XP Cell Signalling Technology | Human | NA | 1:400 | CY3 anti Rabbit 1:200 (Jackson-immuno) |
| Phospho-S3-Cofilin 1 | 3313 (77G2 clone) Cell Signalling Technology | Human | NA | 1:400 | CY3 anti Rabbit 1:200 (Jackson-immuno) |
| DAPI | D1306 ThermoFisher Scientific | NA | NA | 1in100 | NA |
| Phalloidin –Alexa Fluor 633 | A22284 ThermoFisher Scientific | NA | NA | 1in50 | NA |
| Mouse IgG1 | X0931 DAKO | NA | NA | Final Concentration 1ug/ml | NA |
| Rabbit IgG | NB810-56910 Novus | NA | NA | Final Concentration 0.375ug/ml |  |

Table 1 Antibodies, concentration and antigen retrieval conditions for immunohistochemistry. All reagents were from Dako unless otherwise specified. Visualisation was performed using the DAB+ Liquid Substrate Chromogen System (K3467).

**BIMs2A cloning and cell culture experiments.**

pSH570MK was generated from components of a tetracycline controlled plasmid-based expression system [2]. BIMs2A mouse were then subcloned into pSH570MK tetracycline inducible retroviral expression system. Retrovirus was then packaged by transfecting PlatE cells with pSH570MK-BIMs2A or -Empty Vector (EV) using XtremeGene 9 transfection reagent (Roche). MDA-MB-468-EcoR or MDA-MB-231-EcoR (pQCXIN plasmid) breast cancer cells were then infected with filtered viral supernatants and stable cell lines selected using Puromycin. MDA-MB-468 BIMs2A (MDA-MB-468-2A) and MDA-MB-231 BIMs2A (MDA-MB-231-2A) cells were maintained sub-confluent in RPMI complete media (Gibco) containing 10% tetracycline-free FCS and supplemented with 10µg/ml Insulin (media for MDA-MB-231-2A cells was supplemented with 1% HEPES). BIMs2A expression was induced daily with 2 µg/ml Doxycline (DOX) or vehicle control daily in the media and cells were harvested at 72 hours after plating. Annexin V PI staining was performed using the Annexin V-FITC Apoptosis Kit (Biovision, CA USA) as per the manufacturers instructions. IC50 dose curves were performed on each cell line to determine the optimal dose.

**3D organotypic I collagen I/fibroblast invasion assay**

Rat-tail tendon collagen was prepared by extraction with 0.5 M acetic acid to a concentration of ~2 mg/ml. Puromycin-resistant telomerase-immortalized human dermal fibroblasts (TIFs) were prepared by transfecting the empty pSH461MK plasmid using the standard lipfectamine3000 protocol (Thermofisher), prior to 1.0µg/ml puromycin selection for resistant cells. 1x10^5^ cells of these pSH461MK TIFs were embedded in the three-dimensional collagen I matrix. Detached polymerized matrix (2.5 ml) in 35 mm petri dishes was allowed to contract for 14 days. Contracted matrices were seeded with 1x10^5^ MDA-MB-231-2A cells and allowed to grow for 4 days. The matrix was then mounted on a metal grid and raised to an air-liquid interface, which resulted in the matrix being fed from below with media supplemented with either DOX or vehicle control. A second condition was set up so that DOX or vehicle control supplemented media was only added on day 5 of invasion. In both conditions, cells were allowed to invade for a total of 10 days towards the chemo-attractive gradient created by the air liquid interface. **Scoring:** Ten areas of 500x500 μm were selected from 3 independent experiments as representative of the invasion, proliferation or apoptosis for each condition and each antibody. Complete matrix enfoldment and positive multi Cytokeratin staining identified invasive cancer cells. The sum of these cells, and the number of cells on the matrix surface classified as having the ‘opportunity’ to invade, was used to calculate the invasive index as a proportion of invaded cells. The proliferative index was scored as a ratio of brown (Ki67-positive cells) to blue & brown (total cells) within or above the matrix. Similarly, the apoptotic index was scored as a ratio of brown (Cleaved caspase 3-positive cells) to blue and brown (total cells) within or above of the matrix.

**Image analysis**

Quantification of number and size metastasis (using sections stained with an antibody against ant- human-Vimentin) and BIM intensity (using an antibody against anti-human BIM) was performed using a macros designed in FIJI image analysis available at <http://fiji.sc/Fiji> and are available from the corresponding author. Briefly, IHC area analysis was measured by processing 20 images per mouse tumour or lung digitally after acquisition by first applying a tritanope colour blind filter and then replacing red with magenta. A colour deconvolution is applied using the H&E vector to produce a purple, pink, and green RGB image. The purple image is converted to 8-bit grey-scale image and threshold applied, then converted to a binary image, and processed to segment adjacent nuclei. A region of interest (ROI) was used to count and measure the area of all the nuclei. IHC Stain Analysis was performed and the brown image was converted to 8-bit grey-scale image, threshold applied and stain intensity was measured by measuring the grey scale value from 0-255.

**Supplementary References**1. Bradbury JM, Edwards PA, Niemeyer CC, Dale TC: **Wnt-4 expression induces a pregnancy-like growth pattern in reconstituted mammary glands in virgin mice**. *Dev Biol* 1995, **170**(2):553-563.

2. Herr R, Wohrle FU, Danke C, Berens C, Brummer T: **A novel MCF-10A line allowing conditional oncogene expression in 3D culture**. *Cell communication and signaling : CCS* 2011, **9**:17.
