# Supplementary figures and images for: MCL-1 inhibition provides a new way to suppress breast cancer metastasis and increase sensitivity to dasatinib
Source: Breast Cancer Res. 2016 Dec 8;18:125. doi: 10.1186/s13058-016-0781-6 (PMC5146841; doi:10.1186/s13058-016-0781-6)

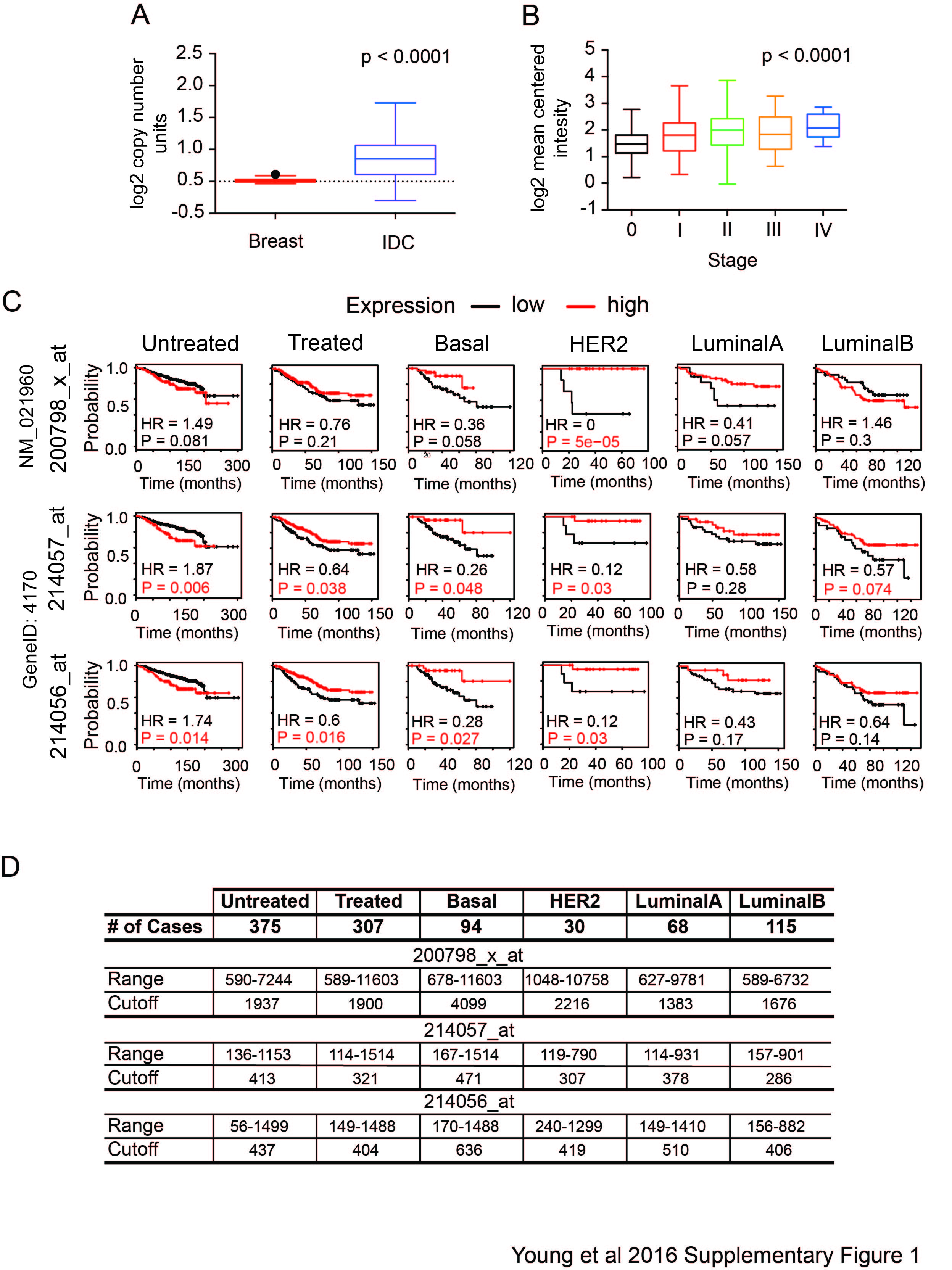

Supplement: Supplementary file 2 — showing MCL-1 expression in human breast cancer. (A) Box–whisker plot depicting the average copy number units (log2) of MCL-1 in invasive ductal breast carcinoma (IDC) in the TCGA2012 cohort: normal n = 111, IDC n = 638. Median, and first and third quartiles indicated by the box, data minimum and maximum points by the bars. (B) Box–whisker plot, format as for (A), depicting the mean centered intensity of MCL-1 mRNA expression in invasive breast carcinoma across stages in the METABRIC dataset. Stage 0 n = 425, Stage I n = 257, Stage I n = 446, Stage II n = 69 and Stage IV n = 8. (C) Kaplan–Meier survival curves and log-rank p values of time to overall survival of patients with triple-negative breast cancer and with MCL-1 CNV alteration. Kaplan–Meier survival curves of overall survival using cases from KM Plotter time using three independent MCL-1 mRNA probes corresponding to Variant 1 (200798_x_at) and full-length MCL-1 (214057_at and 214056_at). Separate Kaplan–Meier survival curves are provided for untreated and treated cases. Treated cases were then split into individual curves split by histopathological subtype. Significant log-rank p values are proved in red and nonsignificant values in black (D) Table depicting the number of cases and the range and optimal cutoff probe values used for each of the Kaplan–Meier curves from (C). (JPG 2111 kb) [file 13058_2016_781_MOESM2_ESM.jpg]

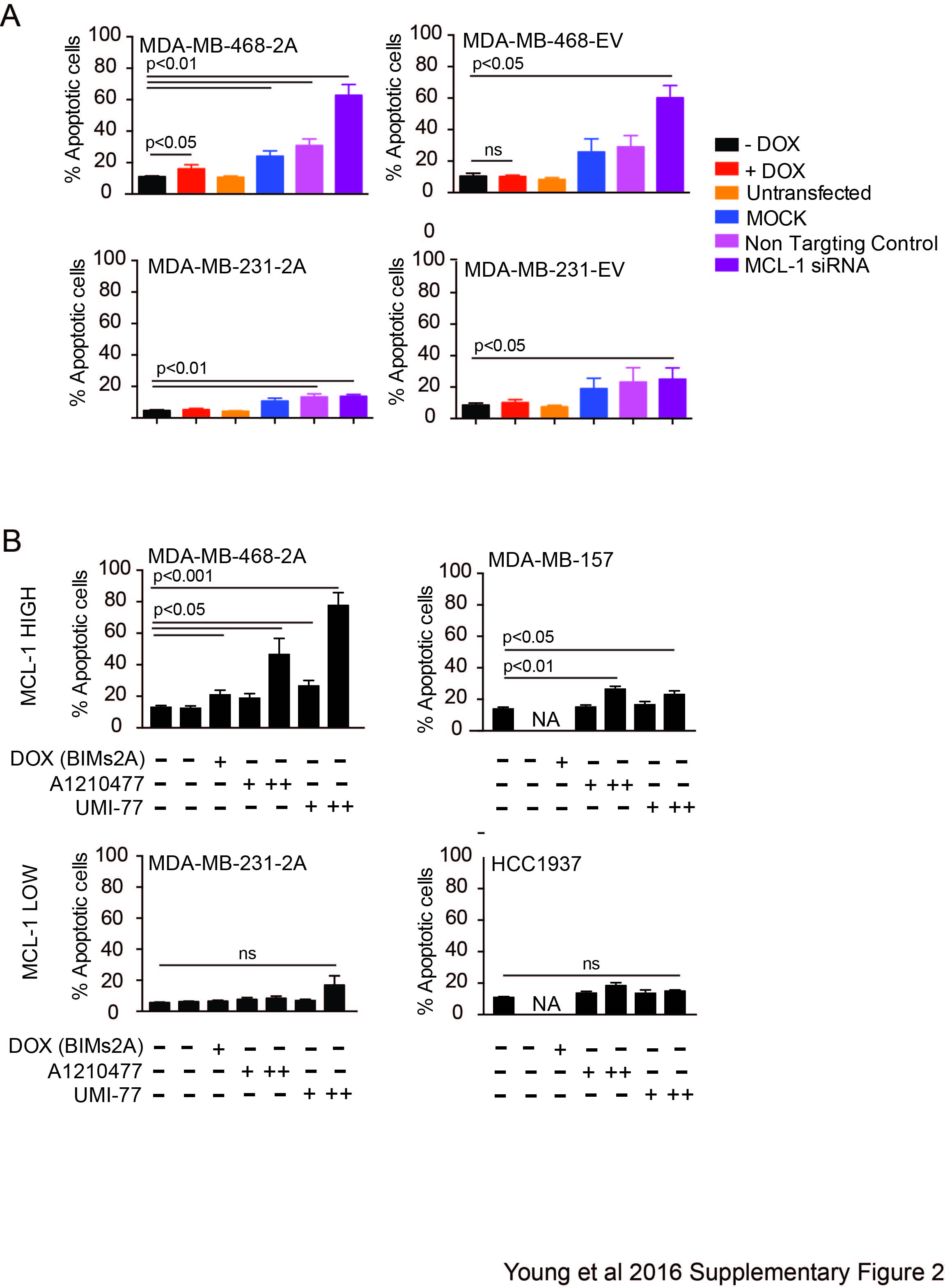

Supplement: Supplementary file 3 — showing the apoptotic effect of MCL-1 siRNA and MCL-1 inhibitors on MDA-MB-468-2A and MDA-MB-231-2A, MDA-MB-157 and HCC-1937 cells. (A) MCL-1 siRNA. Bar graphs depicting the average fraction of apoptotic cells, measured as total Annexin V-positive by flow cytometry, for cells and treatments as indicated at 48 hours. Error bars show standard error of the mean. (B) MCL-1 inhibitors. Bar graphs of apoptosis as measured in (A) for cells and treatments as indicated. For A1210477 and UMI-77: + treated with 5 μM, ++ treated with 10 μM. All graphs are the average of three independent experiments. Bars indicate statistically significant groups, p value unpaired t tests. (JPG 1192 kb) [file 13058_2016_781_MOESM3_ESM.jpg]

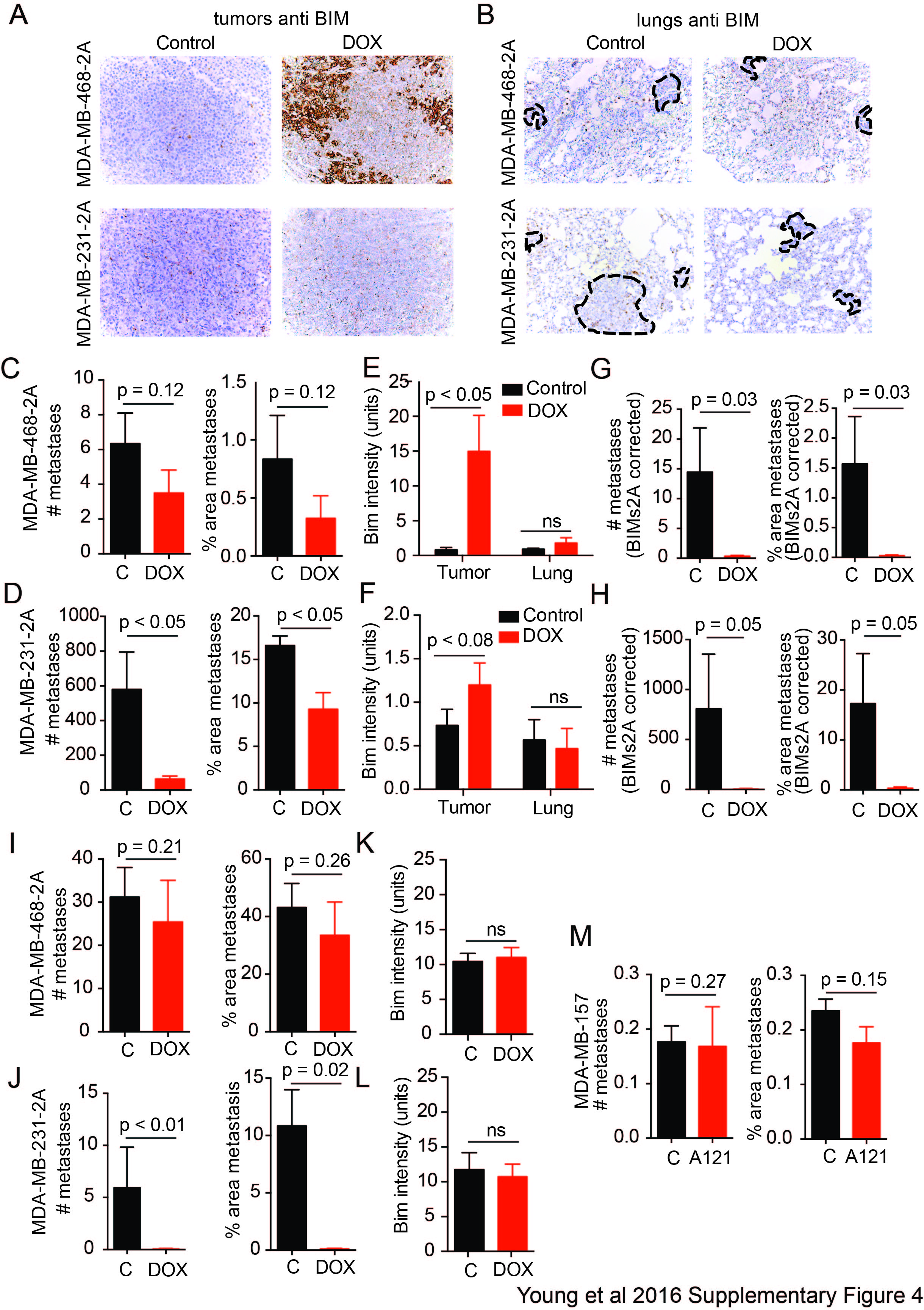

Supplement: Supplementary file 4 — showing BIMs2A expression is induced by DOX in MDA-MB-468-2A and MDA-MB-231-2A xenograft tumors but not induced in the cells from these xenografts that formed the lung metastases. Representative immunohistochemistry images using an antibody to human BIM in the tumors (A) and the lungs (B) of mice bearing MDA-MB-468-2A and MDA-MB-231-2A intraductal xenografts fed DOX or control food. Dotted lines, areas of metastases. Quantitation of the average raw numbers (#) and % area of metastasis in the lungs of mice bearing (C) MDA-MB-468-2A (n = 7) and (D) MDA-MB-231-2A (n = 6) intraductal xenografts after mice were fed DOX or control (C) food. Comparison of the average intensity of human BIM (BIMs2A) in the tumors and the corresponding lung metastases of mice bearing (E) MDA-MB-468-2A (n = 7) and (F) MDA-MB-231-2A (n = 6) intraductal xenografts after mice were fed DOX or control (C) food. Expression of lung metastatic burden relative to the levels of BIMs2A expression in tumors of (G) MDA-MB-468-2A (n = 7) and (H) MDA-MB-231-2A (n = 6) intraductal xenografts. Quantitation of the average number (#) of metastasis and % area of metastasis in the lungs of mice given tail vein injections of (I) MDA-MB-468-2A (n = 10) and (J) MDA-MB-231-2A (n = 10) and given DOX or control (C) food. Quantitation of the average intensity of human BIM (BIMs2A) in the lungs of mice given tail vein injections of (K) MDA-MB-468-2A (n = 10) and (L) MDA-MB-231-2A 2A (n = 10) and given DOX or control (C) food. Quantitation of the average number (#) of metastasis and % area of metastasis in the lungs of mice given tail vein injections of (M) MDA-MB-157 cells and treatment with A1210477 (n = 5) or vehicle control (n = 5). Bars indicate statistically significant groups, Mann–Whitney p value. (JPG 2091 kb) [file 13058_2016_781_MOESM4_ESM.jpg]

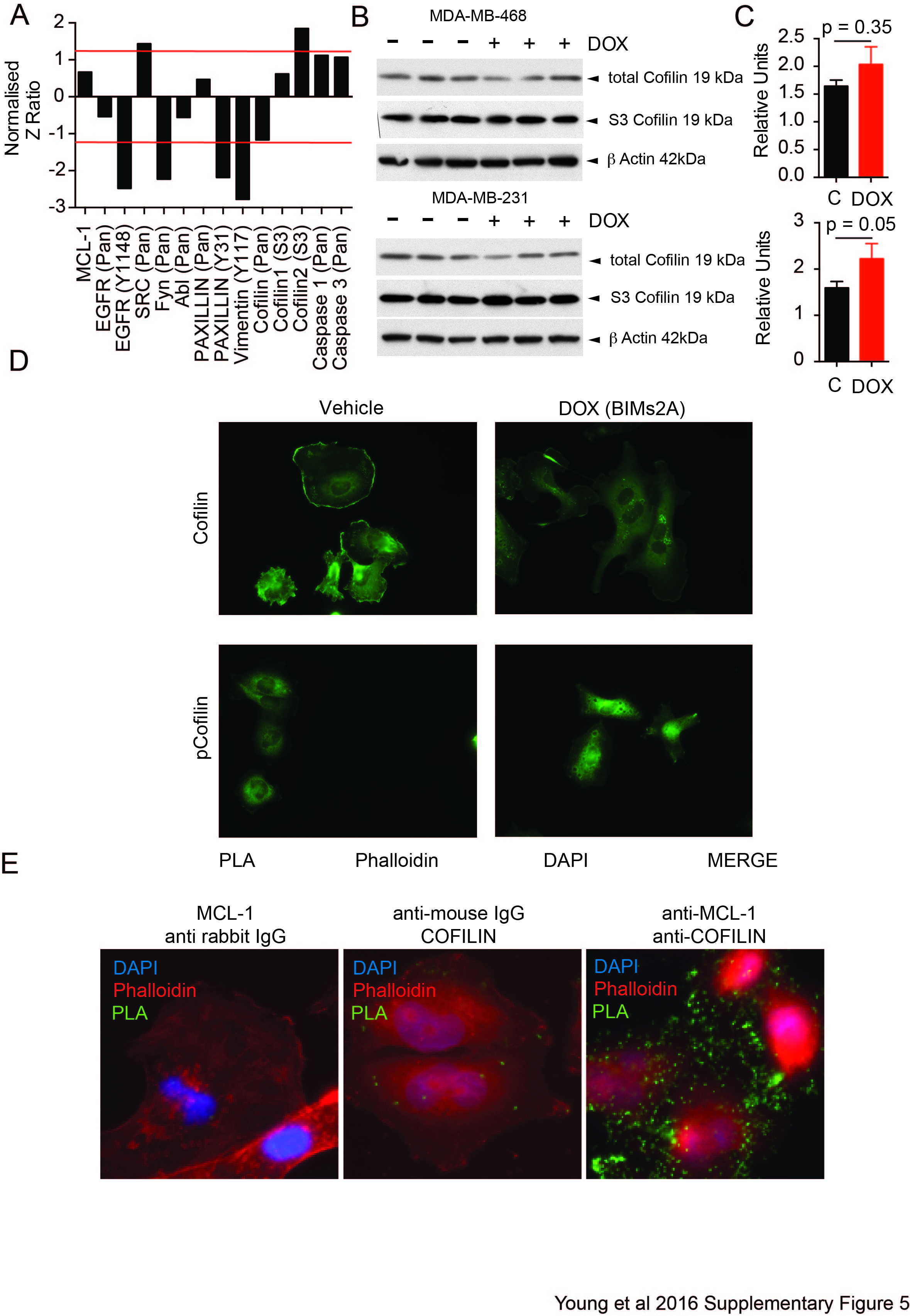

Supplement: Supplementary file 5 — showing that MCL-1 antagonism resulted in changes in proteins involved in SRC family kinase signaling and phosphorylation at serine3 of Cofilin. (A) Normalized z-ratio (a measure of statistical significance) of phosphorylated and total proteins (as indicated) in MDA-MB-468-2A cells at 24 hours after treatment with DOX compared with control cells. (B) Western blots of serine 3 phosphorylated Cofilin, total Cofilin and Actin in xenografts of MDA-MB-468-2A and MDA-MB-231-2A fed DOX food as indicated. (C) Bar graphs depicting the ratio of serine 3 phosphorylated Cofilin to total Cofilin from (B). Bars indicate statistically significant groups, Mann–Whitney p value. (D) Immunofluorescence of Cofilin and p-Cofilin MDA-MB-231-2A cells grown on fibronectin 24 hours after DOX or vehicle treatment. (E) Proximity ligation assays using antibodies to MCL-1 and Cofilin (green), Phalloidin (red) and Dapi (blue) in MDA-MB-231-2A cells. (JPG 1419 kb) [file 13058_2016_781_MOESM5_ESM.jpg]

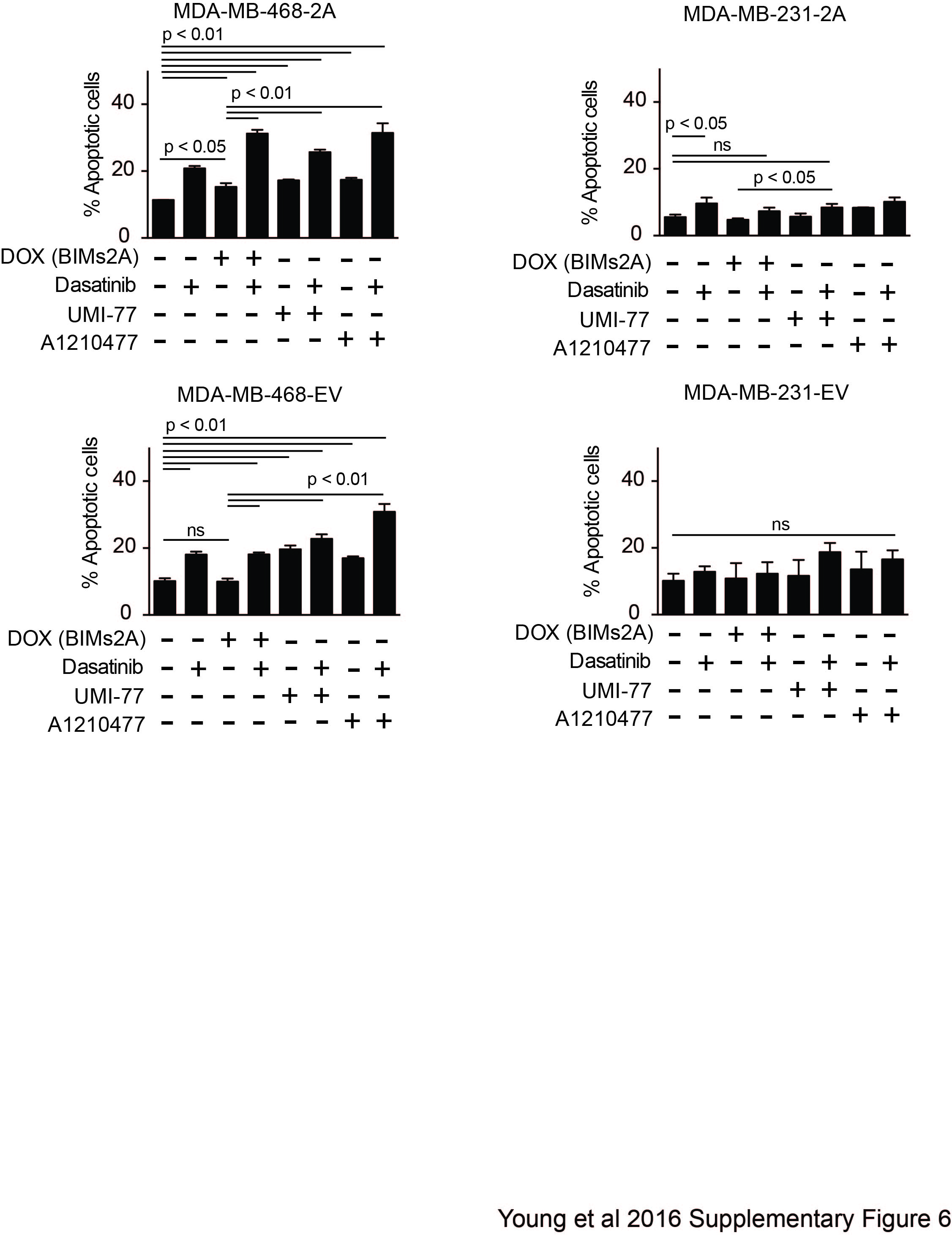

Supplement: Supplementary file 6 — showing that MCL-1 antagonism and Dasatinib treatment induced apoptosis in MDA-MB-468-2A cells but not MDA-MB-231-2A cells when grown in 2D monolayer cultures. Bar graphs depicting the average fraction of apoptotic cells (total Annexin V-positive by flow cytometry) as indicated after 24 hours after treatment with vehicle, DOX, 5 μM A1210477 and 5 μM UMI-77 alone and in combination with 1 μM dasatinib after 24 hours. All graphs and western blots are the average of three independent experiments. Bars indicate statistically significant groups, p value paired t tests. (JPG 1029 kb) [file 13058_2016_781_MOESM6_ESM.jpg]

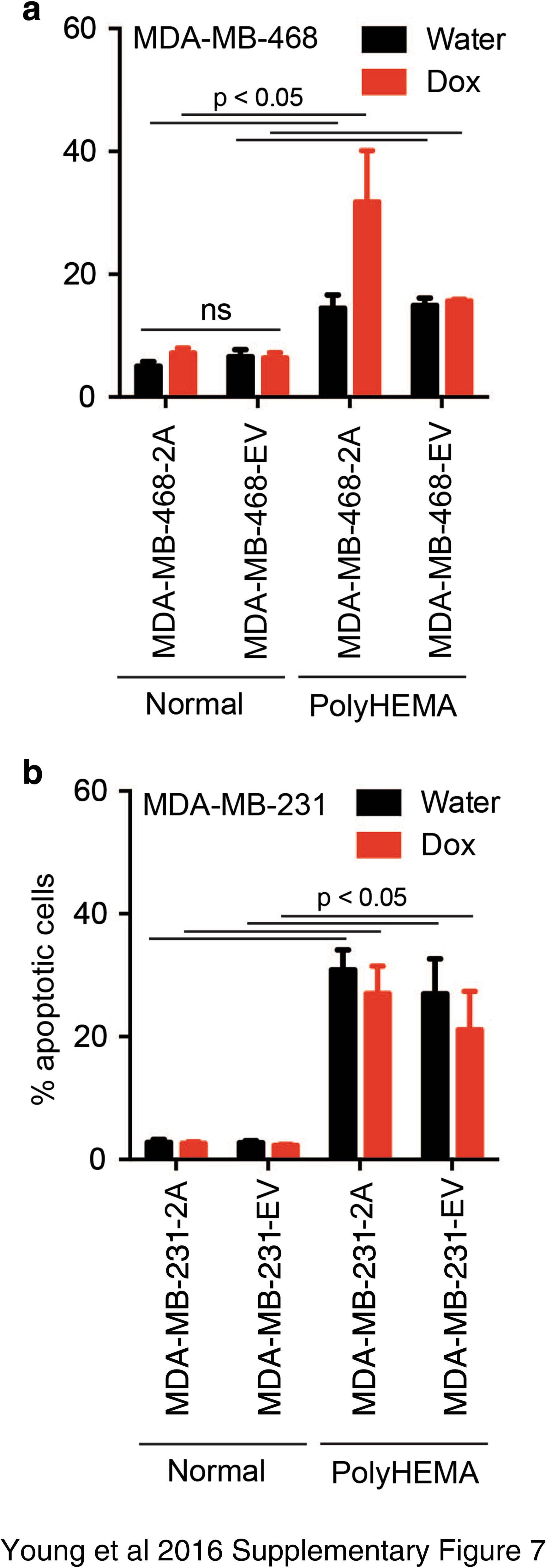

Supplement: Supplementary file 7 — showing that MCL-1 antagonism increased sensitivity to anoikis in MDA-MB-468-2A but not MDA-MB-231-2A cells. Bar graphs depicting the average fraction of apoptotic cells (total Annexin V-positive by flow cytometry) in MDA-MB-468-2A (A) and MDA-MB-231-2A (B) plated as monolayers in culture (normal) or onto PolyHEMA treated plates and harvested at 24 hours after plating. ANOVA p value, dashes indicate statistically significant groups. Bars indicate statistically significant groups, p value paired t tests. (JPG 409 kb) [file 13058_2016_781_MOESM7_ESM.jpg]

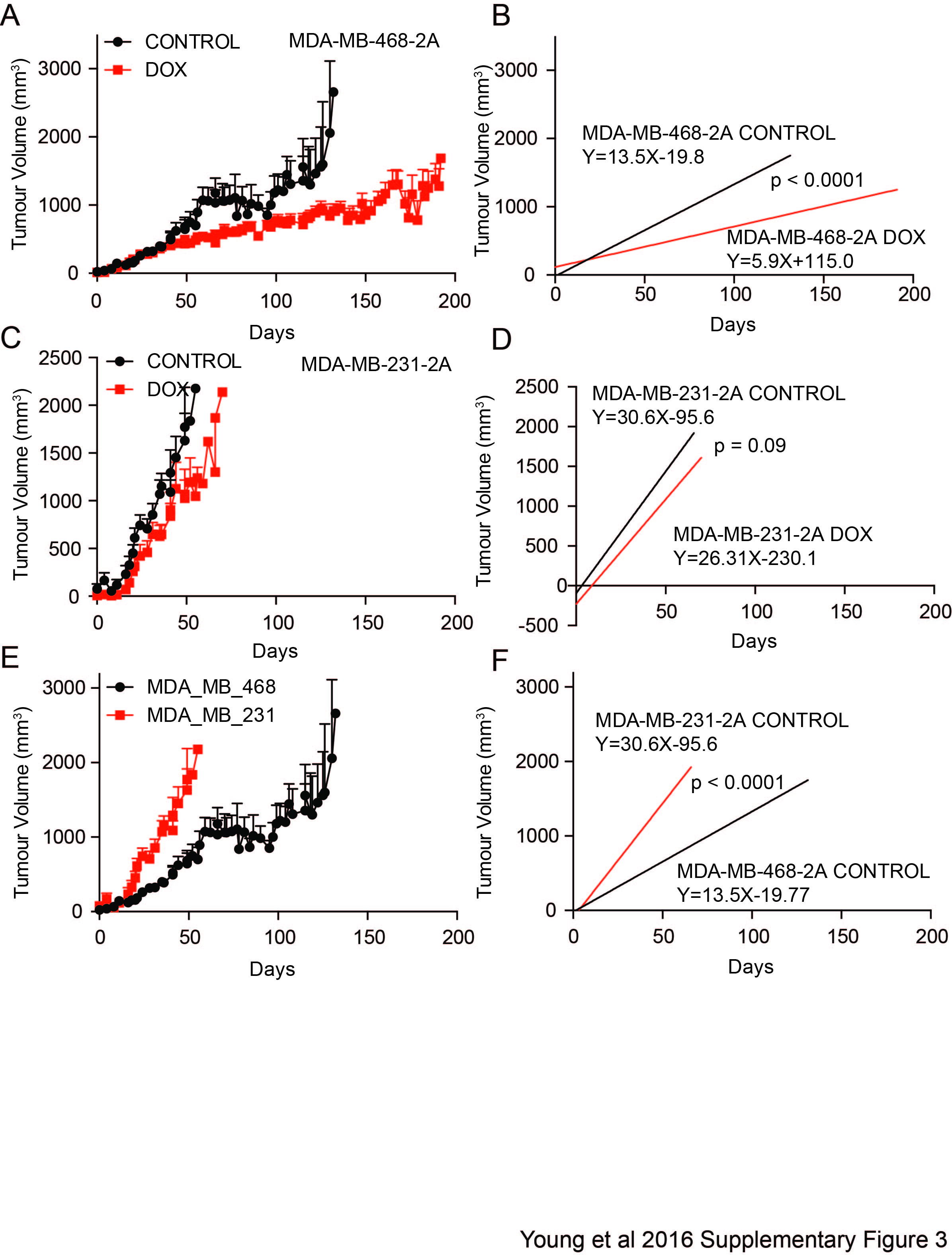

Supplement: Supplementary file 8 — showing that MCL-1 antagonism by BIMs2A slows tumor growth in mice bearing MDA-MB-468-2A xenografts but not MDA-MB-231-2A xenografts. (A–F) Line graphs depicting the tumor growth curves of MDA-MB-468-2A xenografts (A, B) and MDA-MB-231-2A xenografts (C, D) from mice fed with DOX or control food. Linear regression of these curves shown in B and D respectively. A comparison of the growth rate of tumors in mice bearing MDA-MB-468-2A (black) and MDA-MB-231-2A (red) xenografts fed with control food (E, F). (JPG 1348 kb) [file 13058_2016_781_MOESM8_ESM.jpg]
